# Nonviral base editing of *KCNJ13* mutation preserves vision in a model of inherited retinal channelopathy

**DOI:** 10.1172/JCI171356

**Published:** 2023-10-02

**Authors:** Meha Kabra, Pawan K. Shahi, Yuyuan Wang, Divya Sinha, Allison Spillane, Gregory A. Newby, Shivani Saxena, Yao Tong, Yu Chang, Amr A. Abdeen, Kimberly L. Edwards, Cole O. Theisen, David R. Liu, David M. Gamm, Shaoqin Gong, Krishanu Saha, Bikash R. Pattnaik

**Affiliations:** 1 Department of Pediatrics,; 2 McPherson Eye Research Institute,; 3 Department of Biomedical Engineering,; 4 Wisconsin Institute of Discovery, and; 5 Waisman Center, University of Wisconsin–Madison, Madison, Wisconsin, USA.; 6 Merkin Institute of Transformative Technologies in Healthcare, Broad Institute of Harvard and MIT, Cambridge, Massachusetts, USA.; 7 Howard Hughes Medical Institute and; 8 Department of Chemistry and Chemical Biology, Harvard University, Cambridge, Massachusetts, USA.; 9 Department of Ophthalmology and Visual Sciences and; 10 Center for Human Genomics and Precision Medicine, University of Wisconsin–Madison, Madison, Wisconsin, USA.

**Keywords:** Ophthalmology, Gene therapy, Ion channels, Nanotechnology

## Abstract

Clinical genome editing is emerging for rare disease treatment, but one of the major limitations is the targeting of CRISPR editors’ delivery. We delivered base editors to the retinal pigmented epithelium (RPE) in the mouse eye using silica nanocapsules (SNCs) as a treatment for retinal degeneration. Leber congenital amaurosis type 16 (LCA16) is a rare pediatric blindness caused by point mutations in the *KCNJ13* gene, a loss of function inwardly rectifying potassium channel (Kir7.1) in the RPE. SNCs carrying adenine base editor 8e (ABE8e) mRNA and sgRNA precisely and efficiently corrected the *KCNJ13^W53X/W53X^* mutation. Editing in both patient fibroblasts (47%) and human induced pluripotent stem cell–derived RPE (LCA16-iPSC-RPE) (17%) showed minimal off-target editing. We detected functional Kir7.1 channels in the edited LCA16-iPSC-RPE. In the LCA16 mouse model (*Kcnj13*^W53X/+ΔR^), RPE cells targeted SNC delivery of ABE8e mRNA preserved normal vision, measured by full-field electroretinogram (ERG). Moreover, multifocal ERG confirmed the topographic measure of electrical activity primarily originating from the edited retinal area at the injection site. Preserved retina structure after treatment was established by optical coherence tomography (OCT). This preclinical validation of targeted ion channel functional rescue, a challenge for pharmacological and genomic interventions, reinforced the effectiveness of nonviral genome-editing therapy for rare inherited disorders.

## Graphical abstract

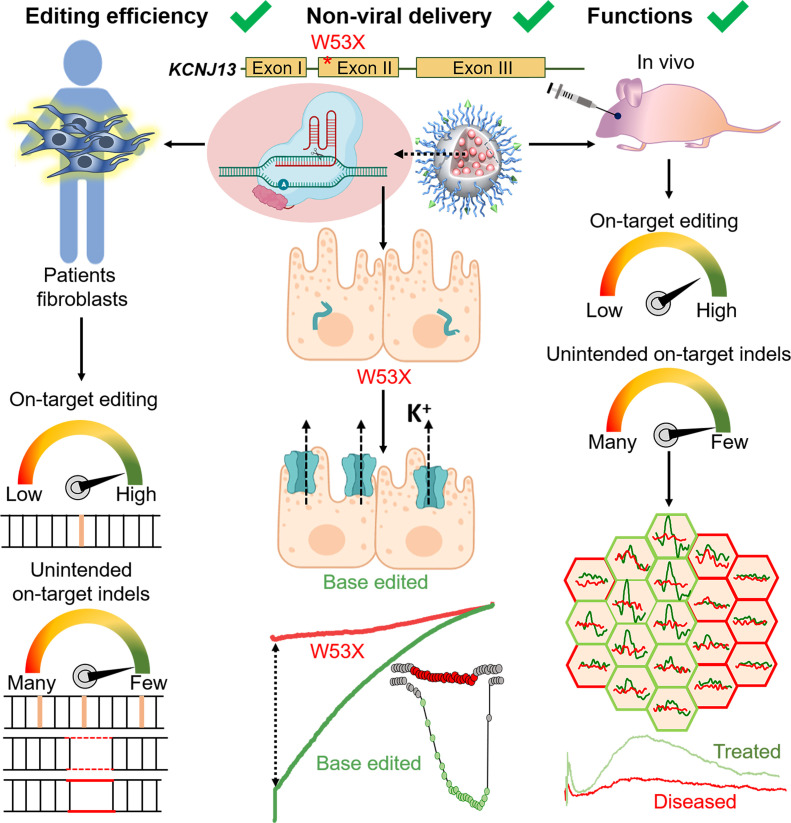

## Introduction

Leber congenital amaurosis type 16 (LCA16; OMIM #614186) is a severe autosomal recessive inherited retinal dystrophy (IRD) leading to blindness in early life. LCA16 is caused by the loss of function of the Kir7.1 potassium ion channel (encoded by the *KCNJ13* gene, location 2q37.1, OMIM #603208) in the apical processes of the retinal pigmented epithelium (RPE) cells of the eye (1, 2). The Kir7.1 channel controls K^+^ homeostasis in the subretinal space, supporting photoreceptor-RPE cell-cell signaling in support of visual functions, such as phototransduction and phagocytosis. Several missense and nonsense loss-of-function mutations have been reported in *KCNJ13*, leading to diminished K^+^ conductance and altered electroretinogram (ERG) of the RPE (3–10).

There is no therapy available for LCA16 patients in the clinic. An LCA16 mouse model with biallelic mutations in *Kcnj13* does not survive long enough to test and develop possible therapeutic strategies. We previously used induced pluripotent stem cells (iPSCs) from a patient with the homozygous W53X mutation in *KCNJ13* to derive RPE cells (LCA16 iPSC-RPE) as a model to test potential treatments such as translational readthrough-inducing drugs (TRIDs) and AAV-mediated gene augmentation (11). However, these possible therapeutics have limitations. TRID-mediated readthrough will broadly affect the proteome and may result in the introduction of nonfunctional amino acids at the site of readthrough. AAV-mediated gene augmentation does not correct the underlying mutations. It may yield only a transient effect alongside the innate and adaptive immune responses that diminishes their therapeutic effect, particularly if repeated doses are required.

In contrast, gene editing could, in principle, correct the endogenous gene and permanently reverse the underlying cause of the disease. However, editing using Cas nucleases is limited by the inefficiency of precise editing via homology-directed repair (HDR) in most retinal cell types (12–14). Since HDR is inefficient in nondividing cells such as the RPE, most edited cells will acquire new indel mutations, and cells with 1 corrected allele frequently coinherit a disruptive indel allele. Moreover, double-strand DNA break (DSB) formation at the target site would pose the risk of uncontrolled indels and other undesired cellular consequences, such as chromosomal translocations, large deletions, aneuploidy, p53 activation, chromothripsis, or transposon insertions (15–24). Base editing has the potential to overcome these limitations by capitalizing on the RNA-guided programmability of CRISPR/Cas9 to deliver a base-modification enzyme that can site specifically convert 1 single nucleotide to another (25).

Most base editors (BE) use a Cas9 nickase fused to an engineered or evolved deaminase enzyme (26–28). Adenine base editors (ABEs) install A>G changes, while cytosine base editors (CBEs) install C>T changes, both within a small editing window specified by the sgRNAs (29). Base editing does not rely on HDR and therefore tends to be efficient in nondividing cells. While delivery of the larger base editor effectors can be challenging, nonviral silica nanocapsules (SNCs) provide certain advantages in that they can transiently and cell specifically deliver a variety of payloads, including nucleic acids and proteins, without integrating into the genome, with decent payload-loading content (~10 wt%) and encapsulation efficiency (>90%) for all of the biologics mentioned above (30–32). We recently reported that SNCs decorated with all-*trans*-retinoic acid (ATRA) could achieve tissue-specific delivery to RPE cells via subretinal injection. This provides an unrealized opportunity to explore SNC-mediated base editor delivery for precise gene correction in LCA16 (33).

Here, we explored the ability of an SNC-delivered base editor to correct the endogenous pathogenic W53X *KCNJ13* point mutation in (a) patient-derived fibroblasts, (b) iPSC-derived RPE, (c) heterozygous knockin *Kcnj13*^W53X/+^ mice, and (d) WT allele–disrupted *Kcnj13*^W53X/+ΔR^ mice via subretinal injection. Adenine base editor 8e (ABE8e) substantially outperformed a CRISPR/Cas9 HDR strategy in terms of efficiency and editing precision. SNC-mediated delivery was sufficient to achieve approximately 16% editing of RPE cells in the mouse retina. Moreover, the high editing efficiency of 47% in patient-derived fibroblasts suggests the opportunity to pursue cell therapies using the engraftment of autologous edited and reprogrammed cells. We then demonstrate functional restoration of the Kir7.1 channel in patient-derived iPSC-RPE and in vivo in the LCA16 mouse (*Kcnj13*^W53X/+ΔR^) model, further revealing the potential of CRISPR base editing to preserve vision in LCA16 patients. Our work provides essential proof of concept that base editing via SNC delivery is a viable strategy for preventing the progression of inherited retinal diseases, particularly channelopathies such as LCA16.

## Results

### ABE8e mRNA efficiently corrects the HEK^W53X/W53X^cell line.

**Figure 1:**
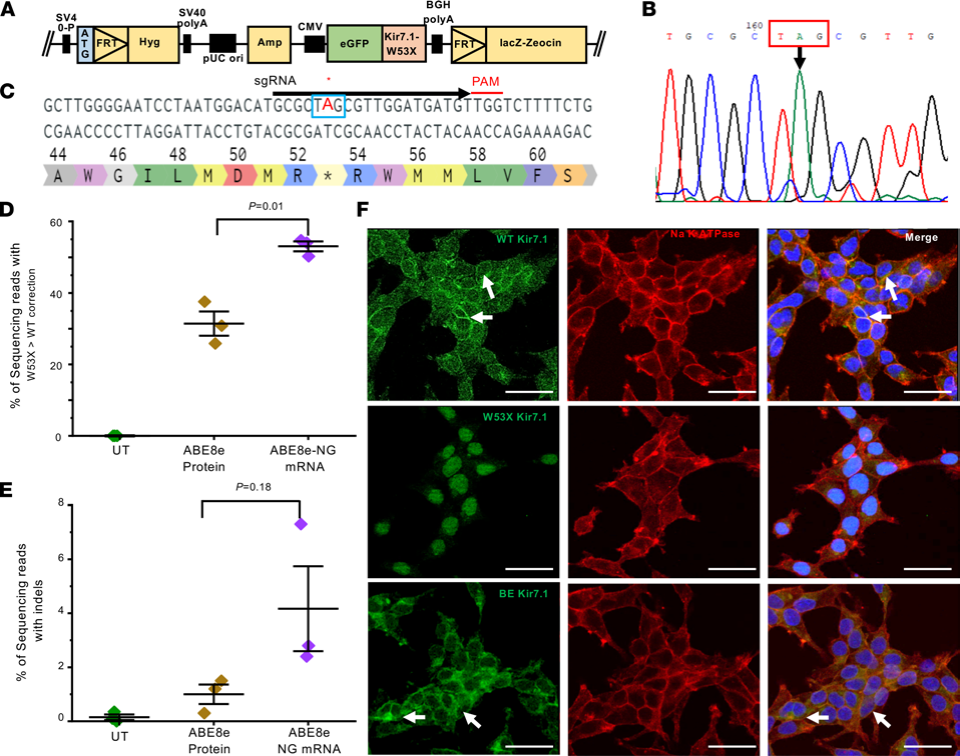
Evaluation of ABE8e RNP and ABE8e mRNA to correct *hKCNJ13*^W53X/W53X^ allele in HEK293 FRT stable cells. (**A**) Construct design to generate HEK293 FRT stable cells harboring the *KCNJ13* ^W53X^ allele. (**B**) Chromatogram generated from HEK293 FRT stable cells showing the W53X codon marked in the red box and the downward black arrow indicating the specific nucleotide change (G>A).(**C**) Schematic of the *hKCNJ13* locus highlighting the mutation c.158G>A (blue box marked with asterisk) and position of the W53X targeting sgRNA (black line) with TGG PAM (red line). (**D**) Base-editing efficiencies are shown as the percentages of sequencing reads with the corrected WT allele (and no other silent changes, bystander edits, or indels) in HEK293^W53X^ cells following electroporation of ABE8e protein+sgRNA (RNP) or ABE8e mRNA+sgRNA (*n* = 3). Markers (diamonds) represent the individual biological replicates (*n* = 3), and error bars represent SEM by 2-tailed Student’s *t* test. (**E**) Percentages of sequencing reads with indels in ABE8e RNP– and ABE8e mRNA–treated stable cells (*n* = 3). Markers (diamonds) represent the individual biological replicates (*n* = 3), and error bars represent SEM by 2-tailed Student’s *t* test. (**F**) Kir7.1 expression in ABE8e mRNA–treated cells assessed by immunocytochemistry. GFP primary antibody was used to enhance the endogenous signal. DAPI was used to stain the nucleus. Scale bars: 50 μm. White arrows mark membrane localization in cells.

Given the relatively low efficiency and heterogeneous outcomes of the traditional Cas9 nuclease-mediated gene-editing approach (Supplementary Note, Supplemental Figure 1, A–D, and Supplemental Figure 2, A–H; supplemental material available online with this article; https://doi.org/10.1172/JCI171356DS1), we focused on base editing to correct the W53X mutation. Base editors are engineered proteins that use the programmable DNA-targeting ability of Cas9 to bring a nucleotide base–modifying enzyme to a precise editing window at the target DNA site. We used 3 bioinformatic tools for sgRNA design (see Methods). These guides directed BE, to convert one or more bases, resulting in an altered DNA sequence without requiring DSBs or relying on cellular HDR machinery. Since the c.158G>A nucleotide change causes *KCNJ13* mutation W53X, an ABE-mediated base-editing strategy can potentially correct the mutation through an A>G conversion. To screen ABEs and validate the sgRNAs for their ability to edit this pathogenic locus with functional restoration of the Kir7.1 channel, we generated human embryonic kidney cells that stably express GFP-tagged *KCNJ13*-W53X (HEKW53X/W53X) and an isogenic WT control line (HEKWT/WT) in which the WT *KCNJ13* sequence was inserted into a single Flp-In recognition target (FRT) site at a transcriptionally active genomic locus (Figure 1, A and B). We chose the ABE8e base-editing effector due to its high efficiency (34, 35) and designed sgRNAs based on the predicted editing window at protospacer positions 4–8, counting the protospacer adjacent motif (PAM) as position number 21–23. Only 1 of the 3 sgRNAs (G*C*G*CUAGCGUUGGAUGAUGU, PAM; TGG) was predicted to have high specificity while also placing the c158G>A position within the editing window of ABE8e (Figure 1C).

We observed 99.7% (99.7% ± 0.1%) transfection efficiency in HEK293 cells by electroporation of 3 μg of GFP mRNA (Supplemental Figure 3, A–C). Therefore, we tested the activity of ABE8e in mRNA and protein formulations via electroporation. HEK^W53X^ cells were electroporated with either a mixture of ABE8e mRNA and sgRNA or ABE8e:sgRNA ribonucleoprotein (RNP) complexes. Deep-sequencing analysis was performed on the treated cells by isolating RNA instead of DNA to distinguish the inserted allele from the endogenous allele of the HEK genome. The pool of electroporated HEK^W53X^ cells showed significantly higher AT-to-GC correction efficiency with ABE8e mRNA (53.0% ± 1.4%) compared with ABE8e RNP (31.4% ± 3.4%) (Figure 1D). Our on-target analysis showed comparatively lower indels introduced using RNP (1.0% ± 0.4%) than mRNA (4.2% ± 1.6%) at and around the protospacer (Figure 1E).

The W53X nonsense mutation in *KCNJ13* disrupts its protein expression in HEK^W53X^ cells, so we assessed whether base editing restored Kir7.1 expression and subcellular localization at the protein level. Immunocytochemistry demonstrated that the Kir7.1 protein is present in the membrane in most of the base-edited HEK^W53X^ cells, similar to control HEK^WT^ cells. On the other hand, the fluorescence signal in untreated HEK^W53X^ cells is accumulated in the cytoplasm and nucleus due to its premature truncation (Figure 1F). These results confirmed the successful translation and trafficking of full-length protein after editing (Figure 1F).

**Figure 2:**
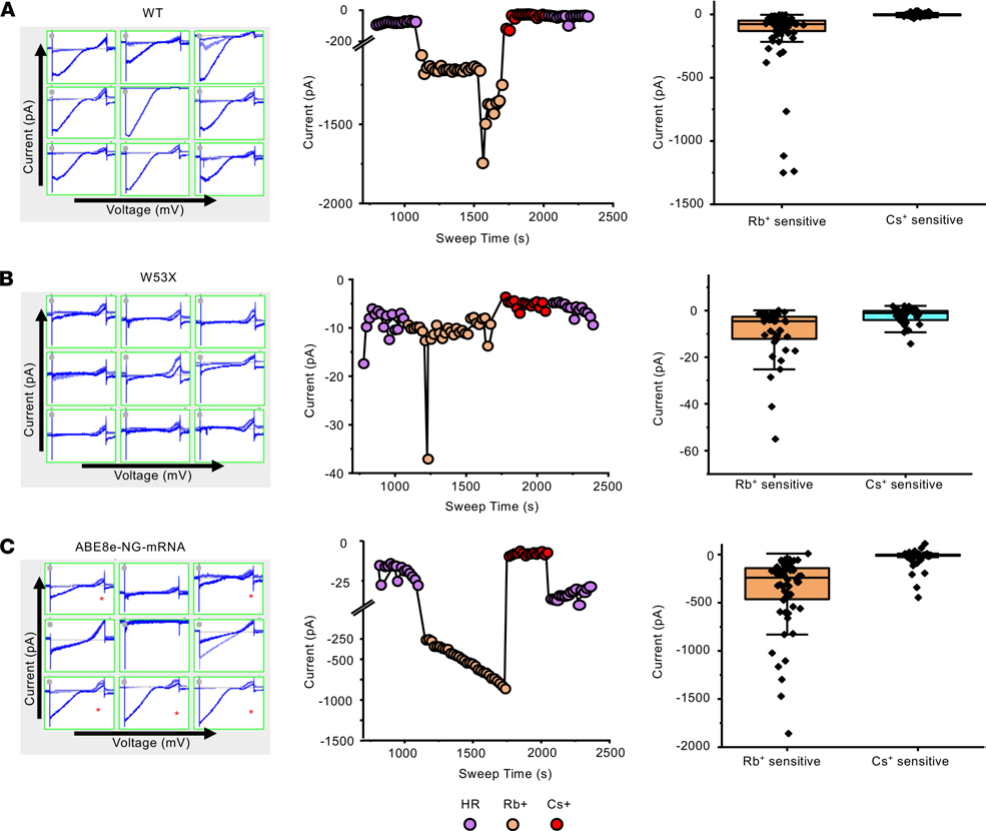
Kir7.1 current recording in WT, W53X, and base-edited W53X HEK293 FRT stable cells. (**A**) Left: snapshots of Kir7.1 current profile in WT stable cells. Center: current-sweep plot represents the experimental timeline and is shown for 1 representative cell. Right: Rb^+^- and Cs^+^-sensitive current in HEK^WT^ stable cells (**B**) Left: snapshots of Kir7.1 current profile in HEK^W53X^ stable cells. Center: current-sweep plot is shown for 1 representative cell. Right: Rb^+^- and Cs^+^-sensitive current in HEK^W53X^ stable cells. (**C**) Left: snapshots of Kir7.1 current profile in HEK^W53X^ base-edited cells using ABE8e mRNA. Cells marked with asterisks showed recovery of K^+^ channel functions after base editing. Center: current-sweep plot is shown for 1 representative cell. Right: Rb^+^- and Cs^+^-sensitive current in HEK^W53^ base-edited cells.

To determine Kir7.1 channel function, whole-cell currents were recorded using an automated patch-clamp system from pools of either base-edited HEK^W53X^ cells, untreated HEK^W53X^ mutant cells, or untreated HEK^WT^ cells (Figure 2, A–C). In a standard external physiological HEPES Ringer (HR) solution, HEK^WT^ cells had a considerable negative membrane potential (–74.2 ± 4.2 mV, *n* = 3 individual cells) (Supplemental Figure 4A). They exhibited an inward rectifying average Kir7.1 current of –45.0 ± 11.3 pA (*n* = 62 individual cells) at –150 mV in 5 mM K^+^. The inward current increased in response to external 140 mM Rb^+^ (a known activator of the Kir7.1 channel) by 4.5-fold (–204.9 ± 37.3 pA) (Supplemental Figure 4B). However, the current was not blocked by adding 20 mM Cs^+^ (a known blocker of the Kir7.1 channel) to the bath solution (–41.5 ± 11.2 pA), suggesting a low Cs^+^ sensitivity of the Kir7.1 channel (Figure 2A), as reported earlier (1, 34). These responses were not observed in untreated HEK^W53X^ mutant cells, with a significantly lower resting current amplitude (–9.7 ± 1.3 pA) and negligible change upon adding Rb^+^ or Cs^+^ (Figure 2B). Importantly, base-edited HEK^W53X/W53X^ cells exhibited restored Kir7.1 function in 80% of cells (*n* = 60) and the characteristic Rb^+^-responsive K^+^ current (–33.3 ± 7.7 pA baseline; –415.2 ± 54.0 pA with Rb^+^, –42.2 ± 9.6 pA with Cs^+^) (Figure 2C). The K^+^ current profile of 20% of the treated HEK^W53X^ cells (*n* = 15) was comparable to that of mutant HEK^W53X/W53X^ untreated cells (*n* = 40), which we attribute as unedited cells (K^+^ current in HR; –30.9 ± 8.0 pA, Rb^+^; –57.7 ± 12.5 pA, Cs^+^; –13.9 ± 5.1 pA) (Figure 2C). From these experiments, we conclude that ABE8e mRNA plus sgRNA delivery can correct the W53X mutation at high efficiency (around 50% of total alleles and in 80% of cells), restoring Kir7.1 protein levels and K^+^ conductance.

### Efficient ex vivo base editing in patient-specific fibroblasts using nanoparticles.

**Figure 3:**
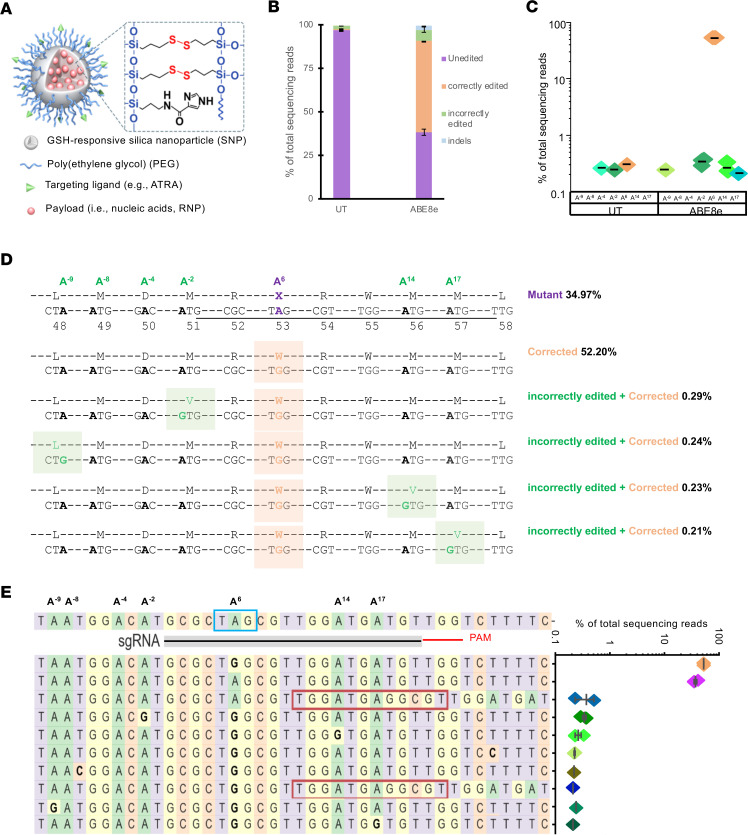
Evaluation of ABE8e mRNA+sgRNA combinations to correct the W53X allele in LCA16 patient fibroblasts. (**A**) Design of SNCs used to encapsulate ABE8e mRNA and sgRNA. (**B**) Base-editing efficiencies are shown as the percentages of total DNA sequencing reads, classified as unedited, correctly edited, or incorrectly edited due to bystander A edits, and with indels in treated and untreated (UT) cells. (**C**) Percentage editing of the target (A^6^) and bystander (A^–9^, A^–8^, A^–4^, A^–2^, A^14^, A^17^) A to G by ABE8e mRNA as observed in 3 independent experiments. (**D**) Amino acid conversion at the respective location was generated due to target and bystander edits. The protospacer sequence is underlined, the pathogenic early stop codon is in a purple box, the target A>G edit is marked in orange, and bystander A edits are in green. (**E**) The sgRNA location is marked by a black line, PAM is marked by a red line, and mutation is in the blue box. All the A bases within the protospacer are numbered 1–20 based on location. The A bases downstream of the protospacer are numbered from –1 to –9, considering +1 as the first base of the protospacer. The top 10 most frequent alleles generated by ABE8e mRNA treatment show the nucleotide distribution around the cleavage site for sgRNA. Substitutions are highlighted in bold, insertions are shown in the red box, and dashes show deletions. The scatterplot shows the frequency of reads observed in treated cells (*n* = 3 biological replicates). Data from replicates are represented as means ± SEM.

To explore the specificity of ABE at the endogenous human *KCNJ13* locus within patient-specific cells, we encapsulated ABE8e mRNA and sgRNA in SNC-PEG (SNC covalently conjugated with polyethylene glycol [PEG]) nanocapsules (Figure 3A) (33) for delivery into fibroblasts derived from an LCA16 (W53X homozygous) patient (Fibro^W53X/W53X^). Five days after treatment, we assessed base-editing efficiency using deep sequencing. As expected, the activity of ABE8e yielded efficient AT-to-GC editing (52.3% ± 0.1%) at the target W53X site (A^6^). Cells from both the treated and untreated populations (Supplemental Figure 5, A–C) had low levels of other substitutions (or possibly sequencing error) in the amplicon, including bystander A>G and other T>G, T>C, and G>A substitutions, with ABE8e exhibiting 6.5% ± 1.4% unwanted substitutions and untreated Fibro^W53X/W53X^ cells showing 2.6% ± 0.1% unwanted substitutions (Figure 3B). Rare indel outcomes were detected in the ABE8e-treated samples (2.7% ± 0.4%) (Figure 3B). Bystander A>G editing near the target nucleotide was rare in the ABE8e-treated samples (A^14^; 0.3% ± 0.0%, A^17^; 0.1% ± 0.0%) (Figure 3C). We also marked some ABE8e activity upstream of the protospacer at positions A^–2^ (0.3% ± 0.0%) and A^–9^ (0.1% ± 0.1%) (Figure 3C). This bystander editing within and outside the protospacer resulted in silent (L48L) and missense (D50G, M51V, M56V, M57V) mutations (Figure 3D). Although these mutations were observed at a very low frequency (<1%) in Fibro^W53X/W53X^ (Figure 3E), it is possible to employ clonal selection of treated fibroblasts before iPSC generation and select a line that carries no bystander/incorrect edits.

In Fibro^W53X/W53X^, we also assessed the activity of another ABE mRNA (ABE7.10max) that has reduced activity and may reduce bystander mutations (Supplemental Figure 6, A–C). This particular ABE mRNA could be advantageous in cases in which fewer on-target corrections could have a beneficial outcome while avoiding detrimental bystander mutations. Unfortunately, the treated cells showed substantially lower AT-to-GC editing efficiency (14.9% ± 1.7%; *P* = 0.000027) than ABE8e at the target W53X site (A^6^). Undesired editing outcomes were also reduced: bystander editing at A^14^; 0.1% ± 0.1%, A^–2^; 0.1% ± 0.1%, A^–4^; 0.2% ± 0.1%, other observed substitutions (4.7% ± 0.8%), and indels (1.4% ± 0.4%) in the amplicon (Supplemental Figure 6, A–C). These results confirmed that ABEs, particularly ABE8e, can efficiently revert the endogenous W53X nonsense mutation to WT in patient-derived fibroblasts.

### Restoration of Kir7.1 channel function in patient-derived iPSC-RPE.

**Figure 4:**
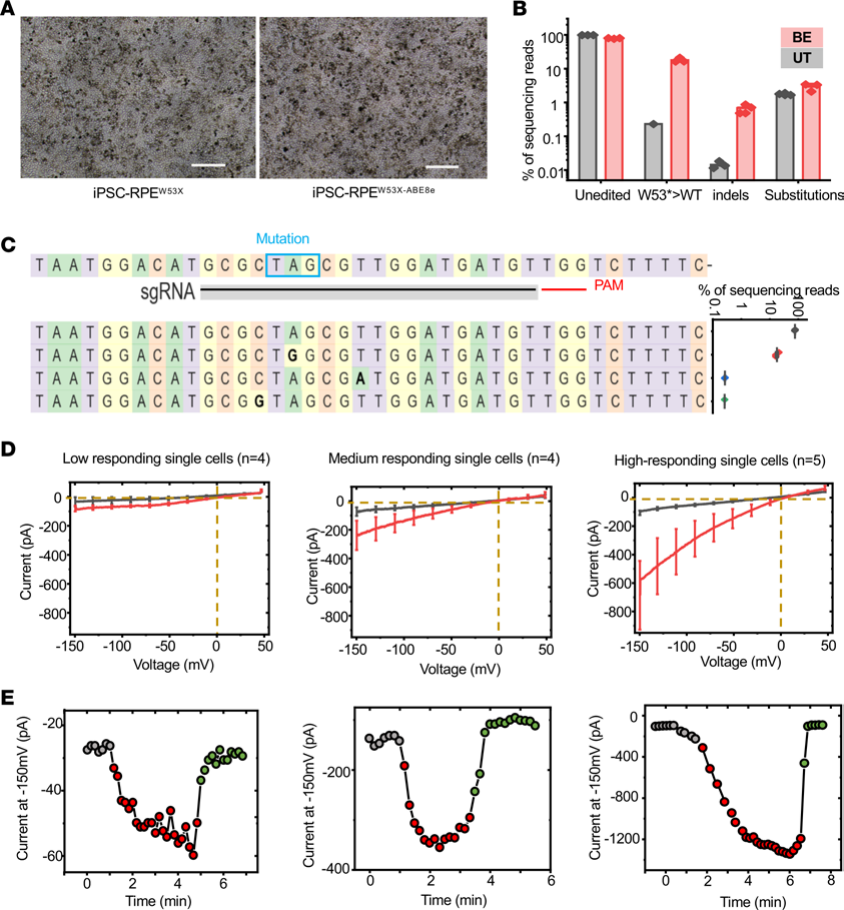
Evaluation of ABE8e to correct W53X alleles in iPSC-RPE^W53X/W53X^. (**A**) Representative bright-field images of base-editor treated and untreated iPSC-RPE^W53X/W53X^. Scale bars: 100 μm. (**B**) Base-editing efficiencies following treatment (BE) with ABE8e mRNA and sgRNA encapsulated in SNC-PEG in iPSC-RPE^W53X/W53X^ as compared with untreated cells. Reads from the untreated and treated cells (*n* = 3) were categorized into 4 subtypes based on their sequences, unedited, W53*>WT, indels, and substitutions. (**C**) Reads generated by ABE8e mRNA treatment showing the nucleotide distribution around the cleavage site for sgRNA. Substitutions are highlighted in bold. The scatterplot shows the frequency of alleles observed in treated cells (*n* = 3). Data are represented as means *±* SEM. (**D**) Manual single-cell patch-clamp assays on iPSC-RPE^W53X/W53X^ cells after treatment with ABE8e. Of the 13 cells assessed for Kir7.1 activity, each could be binned into 1 of 3 classes: low-responding single cells, which appeared to be unedited mutant cells; medium-responding single cells, which showed a low level of Rb^+^ response; and high-responding single cells, which showed Rb^+^ response like WT iPSC-RPE cells. The number (*n*) of cells binned into each class is shown at the top of each graph. (**E**) Current-sweep plot from a representative cell of each bin across a time course of being exposed to physiological HR solution (gray), Rb^+^ stimulation (red), and subsequent wash with HR solution (green).

Next, as a model for in vivo gene-editing therapies for LCA16, we applied our base-editing strategy to patient iPSC-derived RPE harboring homozygous W53X mutation in *KCNJ13*. Previous reports suggest that delivery into RPE by lipofection is challenging. Also, transient expression of BE machinery is preferred over long-term expression via AAVs to restrict off-target editing that may accumulate during prolonged exposure to genome editors. So we used SNC-PEG as a delivery vector for ABE8e mRNA. We observed 27.4% ± 1.7% transfection efficiency in iPSC-RPE cells using SNC-PEG (Supplemental Figure 7D). We did not observe any toxicity in the ABE8e-treated iPSC-RPE^W53X/W53X^, and cell morphology was comparable to that for untreated iPSC-RPE^W53X/W53X^ (Figure 4A). Deep-sequencing analysis in the base-edited iPSC-RPE^W53X/W53X^ revealed 17.8% ± 1.5% editing of the mutant nucleotide to the WT sequence following ABE8e treatment (Figure 4B). Fewer than 4% of resultant alleles carried undesired genomic outcomes: indels quantified in treated RPE had a frequency of 0.7% compared with 0.01% in untreated controls, and bystander substitutions quantified in treated RPE totaled 2.9% ± 0.4% compared with 1.8% ± 0.1% in untreated controls (Figure 4C and Supplemental Figure 7, A–C).

Manual patch-clamp electrophysiology was carried out on the pool of base-edited iPSC-RPE^W53X/W53X^ cells (Figure 4D) to assess the functional rescue of the Kir7.1 channel. Three types of Kir7.1 current profiles were observed in the edited iPSC-RPE^W53X/W53X^ pool. Some of the cells (*n* = 5 out of 13) showed a normal amplitude of K^+^ conductance (–102.0 ± 0.1 pA) in HR solution, which was potentiated in Rb^+^ external solution by 8-fold (–821.0 ± 265.5 pA). These high-responding cells in the pool showed rescue of Kir7.1 channel function and were most likely to have only W53X>WT correction with or without neutral bystander mutations. Some of the cells did not show any recovery of Kir7.1 current (HR; –33.7 ± 31.6 pA, Rb^+^; –80.5 ± 19.4 pA) and had a profile similar to that of iPSC-RPE^W53X/W53X^ cells (11). These low-responding cells (*n* = 4 out of 13) likely experienced no base editing. A few cells (*n* = 4 out of 13) showed slightly higher Rb^+^-stimulated current than the untreated iPSC-RPE^W53X/W53X^ cells, but not as high as iPSC-RPE^WT/WT^ cells. The medium-responding cells showed only a 3-fold Rb^+^ response (–238.7 ± 102.1 pA) for the current amplitude observed in the HR solution (–72.6 ± 29.7 pA). We hypothesize that medium-responding cells contain a relatively low number of ion channels translated from a correctly base-edited *KCNJ13* allele. The current-sweep plots of the representative cells under different treatment solutions are shown in Figure 4E.

### Kcnj13^W53X/+^ monoallelic knockin mouse as an LCA16 model for validating genome editing.

**Figure 5:**
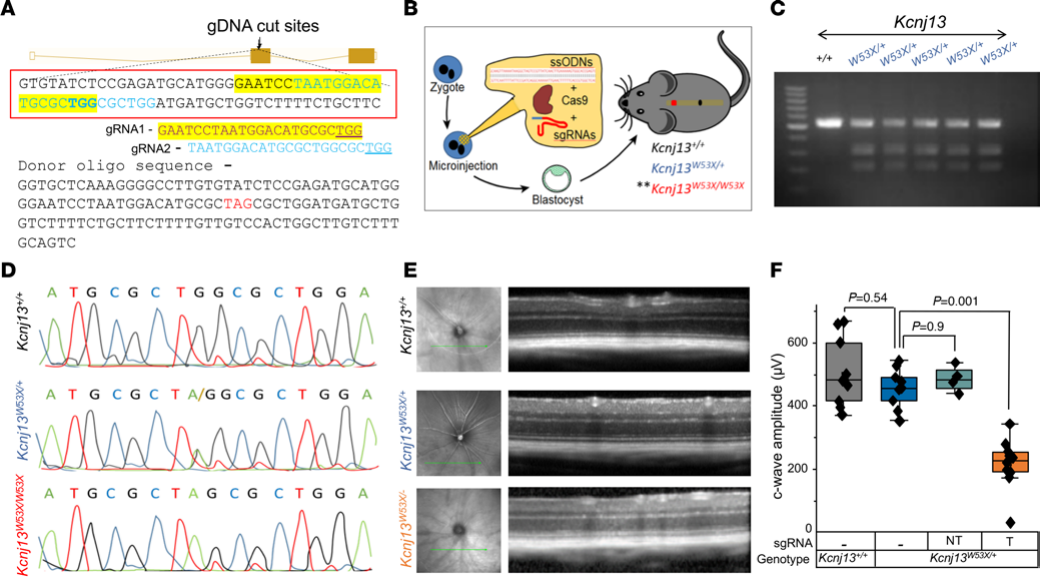
Visual function of *Kcnj13*^W53X/+^ mice is similar to that of *Kcnj13*^+/+^ mice. (**A** and **B**) Two different sgRNAs targeting the *Kcnj13* gene at exon 2 and a ssODN sequence with the desired nucleotide change to generate the *Kcnj13*^W53X/W53X^ mouse model by CRISPR/Cas9 and HDR genome-editing technique by microinjecting them into the pronuclei of the zygote. Double asterisks indicate postnatal day 1 lethal. (**C**) RFLP analysis of the *Kcnj13* gene from the generated mice digested with Nhe1 enzyme on 2% agarose gel. (**D**) Chromatograph confirming the mouse genotype. (**E**) OCT images showing comparison between *Kcnj13*^+/+^, *Kcnj13*^W53X/+^, and WT allele–disrupted *Kcnj13*^W53X/+ΔR^ mice. (**F**) Averaged c wave response confirming WT allele disruption in the RPE of *Kcnj13*^W53X/+ΔR^ using the targeted guide (T). One-way ANOVA with post hoc Tukey’s HSD test was used for comparisons between the groups. NT, nontargeting sgRNA.

A notable limitation to translating our *KCNJ13^W53X^* gene-correction strategy to preclinical models is the lack of a mouse model harboring a homozygous pathogenic mutation in the gene. To test base-editing efficiency in vivo, we first sought to create mice carrying a W53X substitution in the *Kcnj13* gene using CRISPR/Cas9 genome editing. In the pronuclei of zygotes, a combination of Cas9 protein, 2 different specific guides to the *Kcnj13* locus (GAATCCTAATGGACATGCGCTGG and TAATGGACATGCGCTGGCGCTGG), and single-stranded oligodeoxynucleotides (ssODN) was injected (Figure 5, A and B). Five of 6 newborn mice genotyped by sequencing had only 1 allele with the nucleotide alteration. This result was further validated by restriction fragment length polymorphism (RFLP) to digest the PCR product with the NheI enzyme, which selectively digests the mutant allele (Figure 5C). We determined that the phenotype of homozygous mice (Figure 5D) generated from breeding heterozygous founders was lethal on postnatal day one. The retinal structure (optical coherence tomography [OCT] images, Figure 5E) of these heterozygous mice was identical to that of age-matched WT mice. Electrical activity generated by the retina was recorded using ERG as a measure of retinal function in response to a light stimulus. Specific retinal cells generate varying waveforms, a, b, and c waves. The a wave, a negative deflection, corresponds to the photoreceptor. The b wave, which is positive, arises from the inner retinal cells, while the c wave is generated by the RPE cell. Full-field ERG evaluation of heterozygous mice was identical to that of WT mice (Supplemental Figure 8, A–C), and c wave amplitudes measuring RPE cell function (W53X/+; 455.9 ± 18.7 vs. +/+; 504.5 ± 33.4 μV) were not statistically different (*P* = 0.54) (Figure 5F).

**Figure 6:**
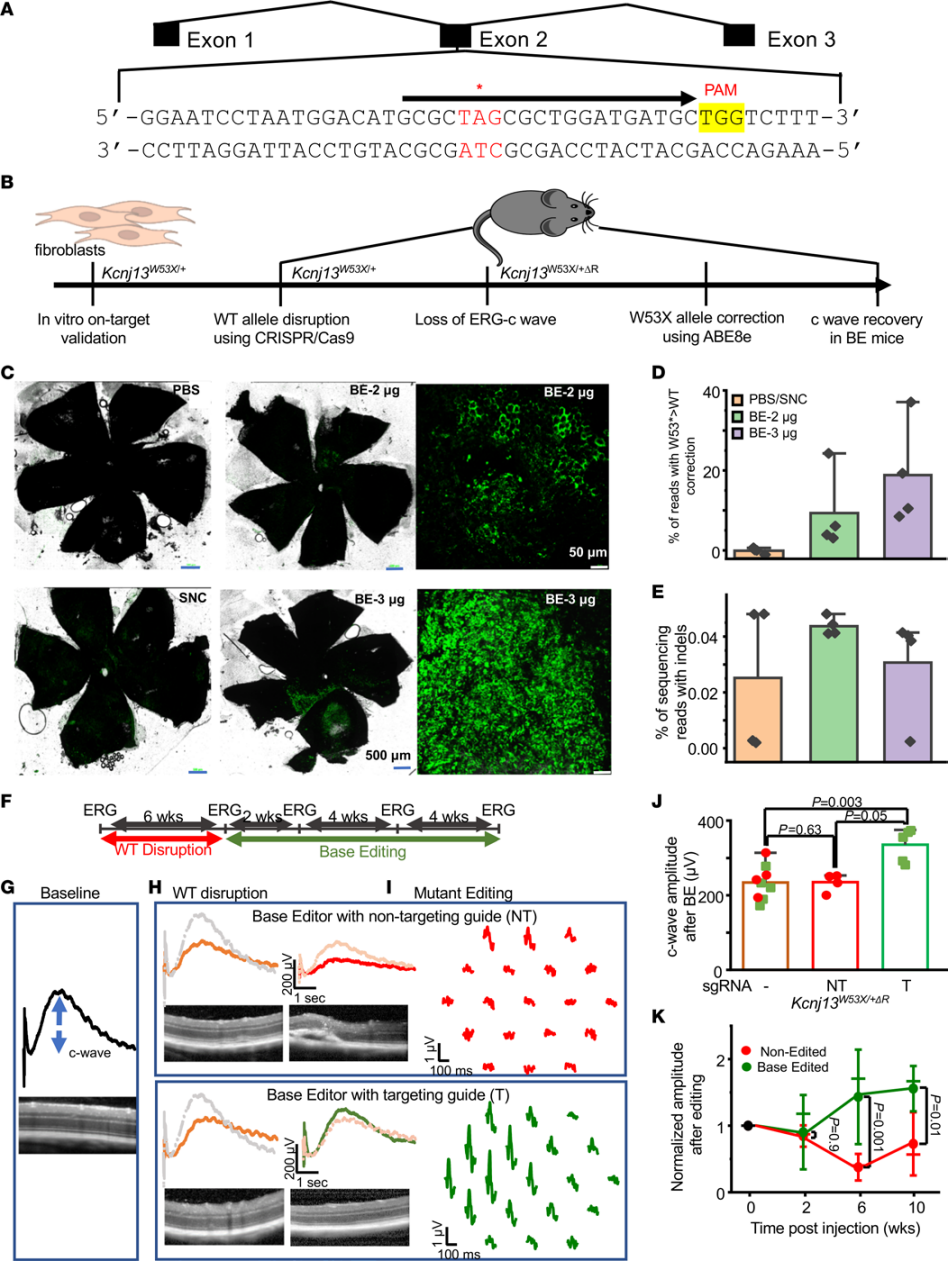
Phenotypic reversal of RPE^W53X^ mice following in vivo ABE8e treatment. (**A**) *Kcnj13^W53X^* allele–specific sgRNA. Black arrow represents the sgRNA spacer sequence, the desired base editing site is indicated by an asterisk, and the PAM is shown in yellow. (**B**) Workflow of in vivo base-editing strategy. (**C**) RPE florets after SNC-PEG-ATRA packaged ABE8e mRNA, W53X sgRNA, and GFP mRNA or empty SNC-PEG-ATRA/PBS as a mock treatment subretinal delivery. (**D**) W53X>WT corrected cell percentages observed in *Kcnj13*^W53X/–^ mice treated with 2 μg or 3 μg of ABE8e. (**E**) Indel percentages observed in *Kcnj13*^W53X/–^ mice treated with 2 μg or 3 μg of ABE8e. (**F**) In vivo experiment time line. Baseline ERG prior to the disruption of the WT allele and after 6 weeks follow-up. ERG prior to injection of the base editor. Recovery monitored for 10 weeks. (**G**) Representation of the c wave amplitude in *Kcnj13*^W53X/+^ mice with retina OCT image. (**H**) Reduced c wave amplitude in the *Kcnj13*^W53X/+^ mice at 6 weeks after disrupting the WT allele with Cas9 protein and WT-specific sgRNA. (**I**) The c wave and mfERG traces following the injection of base editor with a nontargeting guide (red) and base editor with a targeting guide (green). The faded traces represent comparisons before the disruption of the WT allele (gray) and injection of the base editor (orange). (**J**) Average c wave amplitude 6 weeks after the disruption of the WT allele (blue) or after the injection of base editor with a nontargeting guide (red) and targeting guide (green). (**K**) Normalized c wave amplitude in the eyes injected with nontargeting and targeting guides at weeks 2, 6, and 10. One-way ANOVA with post hoc Tukey’s HSD test was used for comparisons between the groups.

We have determined that, unlike in humans, at least 1 normal mouse *Kcnj13* gene allele is required for survival beyond postnatal day 1 (7, 35), precluding the establishment of a genetic model recapitulating the human recessive disease. Therefore, we used our *Kcnj13^W53X/+^* mice to generate an LCA16 mouse model (*Kcnj13*^W53X/+ΔR^) using a Cas9 nuclease complex targeted to disrupt the WT allele (Figure 5, E and F). The WT allele was edited in the RPE cells by delivering Cas9 mRNA and guide (Supplemental Table 9) through subretinal delivery of SNCs decorated with ATRA (SNC-PEG-ATRA), which explicitly targets RPE cells. The size and ζ potential of ABE8e mRNA+sgRNA-encapsulated SNCs are summarized in Supplemental Table 1 and Supplemental Figure 9. Mice were evaluated after 6 weeks to demonstrate intact retinal structure (Figure 6E). ERG measurements of c wave amplitude before the injection were 515.5 ± 9.5 μV, but 6 weeks after the disruption, it dropped to 234.2 ± 14.9 μV (*P* = 0.001). A decrease in c wave amplitude by 54% reflects the functional loss of RPE cells, as in LCA16. This disease model was used for the functional validation of base editing in vivo.

### In vivo base editing via subretinal injection of ABE8e encapsulated SNCs.

We designed sgRNAs targeting the W53X allele (Figure 6A) of mouse *Kcnj13*. We validated it in the fibroblasts derived from *Kcnj13^W53X/+^* mice via electroporation (Figure 6B). To determine the editing efficiency in a heterozygous model system (mouse fibroblasts and in vivo), we delivered using SNC-PEG. We calculated the increase in the WT reads by converting the available W53X allele. We observed a 14.5% increase in the WT reads in the treated fibroblasts, suggesting 29% of cells received an edit (Supplemental Figure 10).

For in vivo subretinal delivery into *Kcnj13^W53X/+^* mice, ABE8e mRNA, sgRNA, and GFP mRNA were packaged into SNC-PEG-ATRA. GFP-positive areas identified the injection site (Figure 6C), and genomic DNA from GFP-positive RPE tissue and the choroid optic cup were used for deep sequencing analysis of the *Kcnj13* gene. We found that delivering 3 μg ABE8e mRNA resulted in 16.8% ± 7.9% RPE editing while delivering 2 μg resulted in 9.5% ± 5.0% RPE editing (*n* = 4 eyes for each dose) (Figure 6D). Our on-target analysis showed no other A>G substitutions outside or within the protospacer region. In addition, indel mutations were not significantly increased over those in placebo-treated mice (Figure 6E). These data demonstrate the specific editing of the W53X allele in vivo by ABE8e-encapsulated SNC-PEG-ATRA.

### Base editing of the LCA16 mutant allele improves RPE function.

In our *Kcnj13*^W53X/+ΔR^ mice, we used ABE8e mRNA, sgRNA packaged in SNC-PEG-ATRA, and administered subretinally to edit the mutant allele. Full-field ERG measurements were performed at weeks 2, 6, and 10 to monitor longitudinal rescue in phenotype (Figure 6F). At week 10, multifocal ERG (mfERG) was performed on injected eyes to determine whether functional recovery was regionalized based on localized delivery of the base-editor complex. For the control group, *Kcnj13*^W53X/+ΔR^ was injected with a base editor and a nontargeting guide.

The nontargeted guide–injected mice exhibited a progressive decrease in c wave amplitude as well as depressed mfERG waveforms in almost the entire retina. On the other hand, the base-edited eyes showed an increase in the c wave amplitude at all measured time points (Figure 6, G–J). mfERG showed localized waveforms (Figure 6H and Supplemental Figure 11A) that perhaps correlated with the site of injection (Figure 6C). On the second week following the injection, the normalized c wave amplitude (to that before the therapy) in eyes receiving the nontargeted guide was 0.8 ± 0.1, practically indistinguishable to that of the eyes injected with the targeted guide, 0.9 ± 0.2 (*P* = 0.9). At 10 weeks after injection, the c wave was reduced (0.7 ± 0.1) for the nontargeted retina compared with an increasing trend (1.6 ± 0.1, *P* = 0.01) (Figure 6K) for the targeted guide–delivered retina. This increase of 55% in amplitude and intact retinal structure, as evaluated by OCT (Figure 6, H and J), demonstrates that inherited retinal channelopathies could be mitigated using base editors. As a further preclinical validation, we determined whether the SNC used to deliver the base editor triggers an immune response. The RPE cells in culture were transduced with either lentivirus, AAV7m8, or AAV5 or were incubated with SNC. Expression of inflammatory cytokines, including IFN-γ, IL-1α, IL-1β, IL-6, CD-8, and Iba1, was assessed by quantitative PCR (qPCR). CD8 and Iba1 showed increased expression in virally transduced RPE cells, but not in cells exposed to SNCs (Supplemental Figure 11B).

### Off-target analysis of sgRNA for human and murine W53X targets confirms specificity.

The Cas9 effector of the ABE dictates the specificity of the base editor to its target site and can bind off-target sites in the genome based on a similarity to the guide sequence. We used an online computational algorithm, Cas-OFFinder (36), to identify the putative off-target sites for the human W53X sgRNA used above in our ABE8e treatments. We found 2,727 potential off-target sites, most harboring 4 mismatches with 1 nucleotide RNA bulge (*n* = 2,004) or DNA bulge (*n* = 512), with other configurations and closer matches occurring much less frequently (Supplemental Figure 12). No off-target site with 2 or fewer mismatches and no DNA/RNA bulge, where off-target editing is most likely to occur, was identified in the human genome for this sgRNA targeted to W53X.

We analyzed the off-target activity of ABE8e in base-edited HEK^W53X/W53X^, LCA^W53X/W53X^, and iPSC-RPE^W53X/W53X^ cells at the 9 potential sites (Supplemental Table 5). Deep-sequencing analysis of these sites showed that ABE8e treatment did not lead to significant off-target A>G editing or introduction of indels compared with that of untreated control cells except at 1 locus. In particular, this 1 off-target (Off_3: GRCh38:50530925 Intergenic) showed 4 times more substitutions in base-edited HEK^W53X/W53X^ cells. However, this nucleotide is already reported in humans to have a natural A>G single nucleotide polymorphism (rs139941105, minor allele frequency in 1000 Genomes = 0.01) of no known clinical importance (Supplemental Figure 13, A and B). These data demonstrate that the off-target modification levels of ABE8e at each of the 9 potential sites are much lower than its on-target editing efficiency and overall indicate a favorable off-target–editing profile.

To detect in vivo off-target editing, we used genomic DNA (gDNA) isolated from the optic cup of base editor–treated heterozygous *Kcnj13*^W53X/+^ mice and screened the 11 top predicted off-target sites by rhAMP-Seq (Integrated DNA Technologies) (Supplemental Table 7). ABE8e treatment introduced on-target W53X>WT correction with an efficiency of 9.5% in these mice. Similarly to what occurred with our in vitro off-target analyses described above, we detected no substantial off-target editing following in vivo editing of mouse RPE (Supplemental Figure 13, C and D). Together, these results suggest that ABE8e could be an effective and specific method to correct polymorphisms in the genome and that SNCs can serve as effective vehicles to correct mutations in the RPE.

## Discussion

Currently, recessive loss-of-function *KCNJ13* point mutations are the only known genetic cause of LCA16, disrupting the Kir7.1 channel function and altering RPE physiology, leading to retinal degeneration with progressive vision loss in patients. In the absence of disease-modifying approved therapies, correction of gene mutations in the RPE cells could result in the avoidance of treatment-related serious adverse events (no off-target responses), restore the Kir7.1 channel function (in vitro and in vivo electrophysiology outcomes), improve vision, or prevent further disease progression (ERG and OCT). Of the 12 different known LCA16-causing *KCNJ13* gene mutations (37), 8 are theoretically accessible by base editors and 5 of those by ABE (requiring A>G [or T>C] edits). Here, we provide essential proof of concept that delivery of ABE8e to RPE via subretinal injection of SNCs can result in correction of the W53X pathogenic allele and restoration of Kir7.1 channel function in RPE cells.

The SNC is a powerful nonviral delivery system for protein and nucleic acids (33). Our group has previously reported SNC-PEG–mediated transfection (50% for plasmid and 60% for mRNA), delivery, and GSH-responsive release characteristics (33, 38). The mRNA transfection efficiency remained unaffected at GSH concentrations lower than 0.5 × 10^−3^ M, suggesting extracellular stability of SNC-PEG (considering plasma/extracellular GSH concentration ranges from 10^−6^ M to 2× 10^−5^ M). However, at GSH concentrations of 0.5 × 10^−3^ M or higher (corresponding to cytosol GSH concentrations between 10^−3^ M and 10^−2^ M), a remarkable decrease in the mRNA transfection efficiency was observed, indicating SNC-PEG can effectively break down in the cytosol to release the payload readily. After treating the cells with the ABE8e mRNA–loaded SNC-PEG, the nanoparticles will stay intact until internalized by the cells. Subsequent to uptake, intracellular GSH will facilitate nanoparticle disassembly and release the encapsulated ABE8e mRNA, enabling targeted base editing within the iPSC-RPE cells. We observed more than 50% on-target editing in patient-derived fibroblasts and 18% in iPSC-RPE cells using the SNC-PEG delivery system. For in vivo editing of mouse RPE, we used the ligand-conjugated SNC-PEG-ATRA and observed 16% of RPE cells were edited. The editing of the RPE cells in vivo is confined, a result of targeted subretinal SNC delivery to localized cells around the injection site. We previously showed that the pathological state of RPE can be reversed by rescuing 25% of the Kir7.1 channel function (11). A recent trial from Editas (Edit101) introduced indels in the *CEP290* gene linked to a different type of LCA, LCA10. It restored visual function in nonhuman primates with more than 10% functional photoreceptors (39). Each RPE sub serves multiple photoreceptors, so 10% RPE correction will rescue many of these photoreceptors. This potential multiplicative effect makes the RPE even more valuable as a target for genome editing. And as with many other ocular diseases, treating LCA16 should not require the correction of all mutant cells. Therefore, the base-editing efficiencies we gained in this study are well within the range of providing considerable therapeutic benefit.

Our study demonstrates an important nonviral delivery method for gene correction in vivo with transient exposure to editing reagents. The ABE activity is anticipated to be transient within cells and on the order of a few hours, considering the half-life of Cas9 mRNA (15 minutes to 3 hours) and the half-life of the sgRNA (less than 3 hours) (40). For our SNC delivered ABE8e to RPE, we have not specifically verified the half-life in our experimental setting, which might be different from what is previously reported. Based on prior studies with transient Cas9 activity, we expect the off-target potential of our nanoparticle treatment to be lower than that of viral-based delivery strategies (41). SNC-mediated base editing can restore Kir7.1 channel function, as assessed in our edited iPSC-RPE cells and in vivo *Kcnj13*^W53X/+ΔR^ mouse studies. Previous studies have demonstrated the functional outcomes of base editing in RPE by correcting a point mutation in the RPE65 enzyme. Base editing restored its enzymatic substrates to support the visual cycle when rescued. Although ion channels such as Kir7.1 catalyze transmembrane flux of ions, their functional restoration has a less established record of success (42). On-target bystander substitutions and indels in some iPSC-RPE and in vivo mouse RPE cells are detectable. Still, they do not interfere with the potential functional benefits of repairing the mutation in the tissue, as these genes are not expressed in the retina. Due to lacking a homozygous LCA16 mouse model, we created an in vivo model by disrupting the WT allele of RPE cells using subretinal delivery of SNC carrying the targeted guide. Our study establishes that in vivo editing with nonviral delivery systems can restore the Kir7.1 channel function in RPE. The edited *Kcnj13*^W53X/+ΔR^ mice displayed recovery of their ERG c wave amplitudes from the edited RPE and had no further retinal degeneration. Additional preclinical validation would require determining safety and efficacy using an acceptable animal model for regulatory clearance.

A limitation of our study is that we do not yet know the clonal editing outcomes of cells edited in vitro or in vivo. The combination of alleles in a single cell is a critical parameter for the function of the tetrameric Kir7.1 channel. Correction of just a single allele, while having detrimental edits in fellow alleles, will likely not preserve RPE health to an extent similar to that of a biallelic correction or monoallelic correction without alteration in the second allele. A clonal analysis would reveal the precise editing outcomes per cell. It could distinguish the phenotypic impact of edited genotypes (43). However, RPE cells from iPSC-RPE and mouse eyes are postmitotic, making clonal amplification of edited cells experimentally intractable in these systems.

In conclusion, while addressing several applicable challenges in correcting an LCA16-causing pathogenic mutation in the Kir7.1 channel, our study provides proof-of-concept therapy for a rare disease. Importantly, base editing of the *KCNJ13^W53X^* allele in vitro and in vivo showed specificity for the W53X mutation without generating detrimental on-target bystander substitutions, indels, or off-target edits elsewhere in the genome. K^+^ conductance in iPSC-RPE in vitro and c wave recovery in *Kcnj13*^W53X/–^ in vivo confirmed the functional rescue of the Kir7.1 channel following base editing. The specific delivery of base editor tools to RPE via the nonviral SNC platform provides a powerful emerging tool for tissue-specific delivery of mRNA or protein-based gene editing therapeutics with good biocompatibility, the ability to package large cargo, increased safety profile, and streamlined manufacturing. These advances will likely direct future preclinical and clinical applications of base editing for correcting mutations causing LCA16 and other ocular genetic diseases.

## Methods

### HEK Flp-In 293 stable cells with GFP-tagged WT and W53X Kir7.1 expression.

HEK Flp-In 293 host cells (Thermo Fisher Scientific, R75007) were generated using a pFRT/lacZeo target site vector to express GFP-tagged Kir7.1 (WT and W53X). These cells contain a single Flp recombination target (FRT) site at a transcriptionally active genomic locus to allow stable integration of the GFP-tagged human *KCNJ13* sequence (WT and W53X). As these cells express the zeocin gene under the SV40 early promoter, complete DMEM high-glucose medium (10% FBS, 1% penicillin-streptomycin [Pen-Strep], 2 mM l-glutamine) containing 100 μg/mL zeocin was used for maintenance. The GFP-WT or GFP-W53X *hKCNJ13* gene sequence was integrated into the genome of these cells based on the manufacturer’s guidelines. Briefly, the cells were cotransfected with FLP-In expression vector (pcDNA5/FRT) containing the GFP-tagged *hKCNJ13* sequence (WT or W53X) created by in-fusion cloning and pOG44 recombinase expression plasmid. The pOG44 plasmid with constitutive expression of the Flp recombinase under the CMV promoter mediates the homologous recombination between the FRT sites of host cells and the expression vector such that the construct from the vector is inserted into the genome at the FRT site. This insertion brings the hygromycin B resistance gene into the frame, inactivates the zeocin fusion gene, and expresses the gene of interest under the CMV promoter. Forty-eight hours after cotransfection, the cells were passaged at 25% confluency to select stable transfectants in 200 μg/mL of hygromycin B. The hygromycin B–resistant cell clones (*n* = 15–20) were picked, maintained in 100 μg/mL hygromycin B, and further expanded for their characterization by genotyping (Sanger sequencing) and protein expression (immunocytochemistry). The primers used for in-fusion cloning and Sanger sequencing are listed in Supplemental Table 2.

### Patient-derived fibroblasts and iPSC-RPE cell culture and maintenance.

Fibroblasts derived from a skin biopsy of an LCA16 patient (7, 11) with homozygous W53X mutation in *KCNJ13* were cultured and maintained in complete DMEM high-glucose medium containing 10% FBS and 1% Pen-Strep at 37°C with 5% CO_2_. iPSCs, reprogrammed from patient-derived fibroblasts, were cultured on Matrigel and differentiated to iPSC-RPE using an approach similar to that previously described (11, 44). Briefly, on day 0 (D0) of differentiation, iPSCs were lifted using ReLeSR (Stem Cell Technologies; catalog 05872) to generate embryoid bodies (EBs). The EBs were maintained overnight in mTeSR^+^ containing 10 μM ROCK inhibitor (R&D Systems; catalog Y-27632). Then, over the next 3 days, the EBs were gradually transitioned to neural induction medium (NIM) (DMEM: F12 1:1, 1% N2 supplement, 1× MEM nonessential amino acids [MEM NEAA], 1× GlutaMAX, and 2 μg/mL heparin; MilliporeSigma). On D7, EBs were plated on Nunc 6-well plates coated with laminin (Thermo Fisher Scientific; catalog 23017015; diluted 1:20 in DMEM/F12). On D16, neurospheres were mechanically lifted. The remaining adherent cells were transitioned to retinal differentiation medium (RDM) (DMEM/F12 [3:1], 2% B27 without retinoic acid, 1% antibiotic-antimycotic solution) and allowed to differentiate to RPE. RDM was supplemented with 10 μM SU5402 (Sigma-Aldrich, catalog SML0443-5MG) and 3 μM CHIR99021 (BioGems, catalog 2520691) for the first 4 medium changes. After more than 60 days of differentiation, iPSC-RPE cells were purified as described by Sharma et al. and cultured on the desired surface (45). Briefly, cultures with differentiated patches of iPSC-RPE were dissociated using 1X TrypLE Select Enzyme (Thermo Fisher, catalog 12563011) and enriched for iPSC-RPE via magnetically activated cell sorting using anti-CD24 and anti-CD56 antibodies (Miltenyi Biotec). iPSC-RPE cells in the negative cell fraction were seeded on the desired surface precoated with laminin (Thermo Fisher Scientific, catalog 23017015; diluted 1:20 in DMEM/F12) and cultured.

### SNCs for adenine base editing and CRISPR/Cas9 gene editing.

We recently reported a safe and efficient nanoplatform, SNC, for delivering CRISPR gene-editing and base-editing components (33). SNCs were synthesized using a water-in-oil microemulsion method. The oil phase (1 mL) was prepared by mixing Triton X-100 (885 μL) with hexanol (0.9 mL) and cyclohexane (3.75 mL). An aliquot of aqueous solution (25 μL) containing the desired payload (ssODN or base editor mRNA+sgRNA, with a total nucleic acid concentration of 2 mg/mL) was mixed with the silica reagents: tetraethyl orthosilicate (TEOS) (4 μL), bis(3-(triethoxysilyl)propyl)-disulfide (BTPD) (6 μL), and N-(3-(triethoxysilyl) propyl)-1H-imidazole-4-carboxamide (TESPIC, 1 mg). The synthesis of TESPIC was reported previously (33). This mixture was homogenized by pipetting and then added to the oil phase (1 mL). The water-in-oil microemulsion was formed by vortexing for 1 minute. Under vigorous stirring (1,500 rpm), an aliquot of 30% aqueous ammonia solution (4 μL) was added, and the water-in-oil microemulsion was stirred at 4°C for 12 hours to obtain unmodified SNCs. Acetone (1.5 mL) was added to the microemulsion to precipitate the SNCs. The precipitate was recovered by centrifugation (15,000*g*) and washed twice with ethanol and 3 times with ultrapure water. The purified SNCs were collected by centrifugation (15,000*g*). The as-prepared, unmodified SNC was redispersed in ultrapure water (1 mL). For surface modification, methoxy-poly (ethylene glycol)-silane (mPEG-silane) or a mixture of mPEG-silane and amine-poly (ethylene glycol)-silane (NH2-PEG-silane, Mn = 5000) (molar ratio of mPEG-silane: NH2-PEG-silane = 8:2) was added to the SNC suspension mentioned above for the synthesis of SNC-PEG without ATRA (i.e., SNC-PEG) or SNC-PEG-NH_2_ (for ATRA conjugation), respectively. The total amount of PEG was 10 wt% of SNC. The pH of the suspension was adjusted to 8.0 using a 30% aqueous ammonia solution. The mixture was stirred at room temperature for 4 hours. The resulting SNCs were purified by washing with ultrapure water 3 times and concentrated with Amicon Ultra Centrifugal Filters (MilliporeSigma). ATRA was conjugated onto SNC-PEG-NH_2_ via 1-ethyl-3-(3-dimethylaminopropyl)carbodiimide (EDC)/*N*-hydroxysuccinimide (NHS) catalyzed amidation. Payload-encapsulated SNC-PEG-NH_2_ (0.5 mg) was redispersed in 1 mL DI water. EDC (7.5 μg), NHS (4.5 μg), and a DMSO solution of ATRA (6 μg in 5 μL DMSO) were added to the above solution. The solution was stirred at room temperature for 6 hours, and then the resulting ATRA-conjugated SNC (i.e., SNC-PEG-ATRA) was washed with water 3 times. The SNC-PEG-ATRA was concentrated with Amicon Ultra Centrifugal Filters to a payload concentration of 2 mg/mL before use. Materials used were as follows: TEOS, Triton X-100, acetone, ethanol, EDC, NHS, and ammonia (30% water) (purchased from Thermo Fisher Scientific). Hexanol and cyclohexane were purchased from the Tokyo Chemical Industry Co. DMSO was purchased from Alfa Aesar. BTPD was purchased from Gelest Inc. mPEG-silane (Mn = 5000) and amine-poly (ethylene glycol)-silane (NH_2_-PEG-silane, Mn = 5000) were purchased from Biochempeg Scientific Inc. ATRA was purchased from Santa Cruz Biotechnology Inc.

### Base-editor mRNAs.

Using their mammalian-optimized UTR sequences, base-editor mRNAs were obtained as a custom product from Trilink Biotechnologies. The mRNAs were synthesized with complete substitution of uracil by N1-methylpseudouridine and cotranscriptional 5′ capping with the CleanCap AG analog resulting in a 5′ Cap1 structure and included a 120 nucleotide polyA tail.

### sgRNA design.

The sgRNAs targeting the W53X location in the human *KCNJ13* gene were designed using Benchling (https://www.benchling.com). The design was validated with 2 other online tools, CRISPR-RGEN (46) and PnB Designer (47), to confirm its on-target specificity (Supplemental Table 8). Only 1 sgRNA (Figure 1C) appeared to be very specific for the W53X location, as it would allow the binding of the spCas9 domain to the target locus that positions c.158G>A site within the editing window of ABE (4–8 for ABE8e, counting the PAM as 21–23). This sgRNA also had the highest on-target score (65.7) and lowest off-target score (56.8) among the Benchling-designed sgRNAs. The sgRNA targeting mouse *Kcnj13* was also selected based on the above criteria (highest on-target score, 57.1; lowest off-target score, 56.8). The chemically modified forms of these sgRNAs (human, G*C*G*CUAGCGUUGGAUGAUGU; mouse, G*C*G*CUAGCGCUGGAUGAUGC) were ordered from Synthego.

### Generation of the lentiviral vector for Cas9-mediated gene editing.

Lentivirus was manufactured for Cas9-mediated editing as described in Gándara, Carolina, et al. (48). Briefly, the HEK293 cells were plated and ready for transfection 24 hours after plating. The cells were transfected with the target plasmid when they reached 70% confluence, along with the packaging gene plasmids pMD2.G and psPAX2. The target vector plasmid contains a copy of the LCA guide, Cas9 protein, and mCherry reporter transgene driven by the EF-1α promoter. Cell culture supernatant was collected from HEK293 after transfection and was concentrated at 1,500*g* for 45 minutes. The concentrated lentivirus titer was between 10^7^ and 10^10^ particles/mL, estimated via functional testing in HEK293 cells.

### Gene editing in iPSC-RPE using lentiviral transduction to deliver Cas9, sgRNA, and SNC to deliver ssODN.

For our attempt to edit the *KCNJ13* gene carrying a W53X nonsense mutation, we used viral transduction to deliver Cas9 and a sgRNA (TAATGGACATGCGCTAGCGT) to the mature iPSC-RPE cells. We used lentiviral vectors (100 MOI) designed explicitly for this purpose: lentiCRISPR v2-mCherry (Addgene plasmid 99154), a gift from Agata Smogorzewska, with the annealed sgRNA oligonucleotides cloned into it using the BsmB1 enzyme digestion and ligation. Successful integration of the sgRNA sequence was confirmed by DNA sequencing with the primer 5′-GGACTATCATATGCTTACCG-3′ for the U6 promoter, which also drives sgRNA expression. Lentivirus was generated in-house using the method described above. This lentivirus was used to transduce mature iPSC-RPE cells to express Cas9, sgRNA, and the reporter gene mCherry, allowing easy identification of transduced cells. After 6 hours of viral transduction, 3 μg of the HDR repair template for W53X gene correction, ssODN-ATTO488 (GATGCTTGGGG**G**ATCCTAATGGA**T**ATGCGCT**G**GCGTTGGATGATGTT**A**GTCTTTTCTGCTTCT; bold letters show the wobble changes), was delivered to the cells using SNC and incubated for 48 hours. Papain digestion was used to dissociate cells that expressed both red and green fluorescent markers, indicating that they had received both Cas9+sgRNA and ssODN constructs, into a single cell that was then used for patch-clamp experiments.

### Base editing in Kir7.1-HEK293 stable cells by electroporation.

HEK293 stable cells expressing *KCNJ13^W53X^*-GFP were subcultured 24 hours before nucleofection at 70% confluency. The ABE8e mRNA (spCas9-NG, 3 μg) (49) and sgRNA (100 pmol) or RNP complex formed by incubating the mixture of ABE8e protein (3 μg) and sgRNA (100 pmol) at room temperature for 10 minutes was introduced via electroporation; 1 × 10^5^ cells were electroporated using the FS-100 program in the Lonza 4D nucleofector according to the manufacturer’s guidelines. After electroporation, cells were seeded in a 6-well plate and maintained in complete DMEM medium containing 100 μg/mL hygromycin B for further analysis.

### Base editing in LCA16-patient’s specific fibroblasts and iPSC-RPE by SNCs.

The W53X-LCA16-patient’s specific fibroblasts (Fibro^W53X^) were subcultured 1 day before treatment. For base editing, ABE8e mRNA (3 μg) and sgRNA (100 pmol) were delivered to fibroblasts using SNCs. Five days after treatment, DNA was isolated for genomic analysis. For base editing in iPSC-RPE, the cells were first seeded in a 96-well plate at a density of 50,000 cells per well in RDM containing 10% FBS and 10 μM ROCK inhibitor (R&D Systems; catalog Y-27632). On D2, the media was switched to RDM containing 2% FBS. On D3 after seeding, ABE8e mRNA (3 μg) and sgRNA (100 pmol) were delivered to the cells using SNCs in RDM. the iPSC-RPE monolayer was dissociated 2 days after treatment with SNC-ABE8e. Cells were seeded on Transwell inserts and also collected for gDNA analysis. iPSC-RPE cells transitioned to Transwell inserts and were cultured for 4 to 6 weeks to obtain a polarized monolayer of RPE and subsequently analyzed for Kir7.1 channel function by the whole-cell patch-clamp approach. Untreated cells were used as references.

### Generating the Kcnj13^W53X/+^ knockin mouse model.

The *Kcnj13^W53X^* mouse model was generated by Cyagen Biosciences using CRISPR/Cas9-mediated genome engineering. Exon 2 was selected as the target site for the intended base change knockin using 2 distinct guides and a donor sequence. For HDR, the donor oligo carried the mutation p.W53* (TGG to TAG) flanked by 120 bp homologous sequences combined on both sides. Microinjections of Cas9 protein, sgRNA mixture, and ssODN were made into the pronucleus of fertilized eggs. The embryos were then transplanted to the pseudopregnant mice, and the resulting progeny were genotyped using PCR and RFLP to validate the desired gene mutation.

### OCT.

OCT was performed on mice anesthetized with ketamine/xylazine and whose pupils were dilated with 1% tropicamide using the Spectralis HRA+OCT system (Heidelberg Engineering Inc.). The mice were placed on a heating pad that was maintained at 37°C throughout the procedure, and a drop of artificial tear was applied before placing the corneal lens to keep the eyes from drying out. The images were captured and analyzed with Heidelberg Eye Explorer software (version 1.10.0.0).

### ERG in mice.

ERG was performed in mice using a standard protocol described elsewhere before and after the base editing to evaluate the function of the retina. Briefly, the mice were dark adapted overnight prior to ERG. ERG signals were captured in full darkness using an Espion Ganzfeld full-field system (Diagnosys LLC). When using a contact electrode, a drop of 2% hypromellose solution was applied to the eye in order to keep the cornea wet and make electrical contact. Mice were subjected to mfERG testing with the Celeris system (Diagnosys LLC), in which the retina is divided into 19 hexagonal areas and stimulated by a pseudorandom sequence of black and white hexagons that alternate multiple times per second. Data acquired were analyzed with Espion software (Diagnosys LLC; version V6.0.54) and Origin 2018b (OriginLab Corp.). For a and b waves, the eyes were exposed to a series of flash intensities (0.03 to 30 cd.s/m^2^) using a ganzfeld dome for 400 ms with a 2-second interval between flashes. For c waves, the eyes were exposed to 25 cd.s/m^2^ for 4 seconds. Animals were subjected to ERG every 2 weeks.

### Base editing in mice.

C57BL/6J male and female mice (*Kcnj13^W53X/+^*) were housed at the animal facility at University of Wisconsin–Madison under a 12-hour light/12-hour dark cycle at a controlled temperature (25 ± 5°C) and humidity (40%–50%). The mice were genotyped using standard PCR methods with the primers listed in Supplemental Table 3, followed by digestion with restriction enzyme NheI (Anza IVGN0066, Thermo Fisher). The W53X mutation creates a restriction site for NheI, and therefore the W53X allele resulted in 2 (212 bp and 302 bp) fragments compared with only 1 (514 bp) fragment in the WT allele (Supplemental Figure 14). W53X-targeting sgRNA was designed and validated in mouse fibroblasts (50) isolated from *Kcnj13*^W53X/+^ mice. A subretinal injection (2 μL) of SNC-PEG-ATRA encapsulating ABE8e mRNA (2 and 3 μg), W53X-targeting sgRNA (Supplemental Table 9) (100 pmol), and GFP mRNA (1 μg, to visualize the site of injection) was performed in mice (*n* = 4 eyes). PBS or empty SNC-PEG-ATRA (*n* = 4) injected eyes were used as references. Five days after injection, imaging was carried out to assess the delivery based on GFP reporter mRNA. gDNA was isolated from the optic cup of these mice to determine the on-target and off-target effects of base editing. To determine the editing efficiency in this monogenic W53X mouse model at the cellular level, any increase in the WT reads after base editing of the W53X allele was noted and doubled to get the number of cells edited.

### On-target analysis by deep sequencing.

Treated and untreated cells were dissociated using enzymatic treatment (Accutase/Papain) according to the manufacturer’s instructions for genomic analysis. From the HEK293 stable cells, total RNA (QIAGEN, 74134) was isolated, reverse transcribed to cDNA (Thermo Fisher, 4368814), and subsequently amplified for on-target analysis using *KCNJ13* Illumina-specific primers (Supplemental Table 3). From fibroblasts, iPSC-RPE, and mouse optic cups, gDNA was isolated according to the manufacturer’s guidelines (Quick-DNA Miniprep Plus Kit, D4069) and quantified using Nanodrop 2000 or Qubit (Thermo Fisher). For deep sequencing of the *KCNJ13* locus, gDNA was amplified using Illumina-specific primers with adapter sequences (amplicon size, –150 bp) (Supplemental Table 4). Unique indexes (i7 and i5) were ligated to each custom amplicon by PCR (amplicon size, 250 bp), and the indexed amplicons were pooled and purified using AMPure XP beads (Beckman Coulter, A63881). The indexed library was run on an Illumina MiniSeq instrument with a read length of 150 bp. Deep-sequencing data were analyzed using RGEN Cas-analyzer (51) and CRISPResso2 (52) software.

### Off-target analysis by deep sequencing.

The potential off-target sites for the hW53X-sgRNA were identified by an in silico tool, Cas-OFFinder (36). The parameters used were an NG/NGG/NAG PAM with up to 4 mismatches to the sgRNA sequence (Supplemental Table 5 and Supplemental Figure 12). We also considered a DNA and RNA bulge of 1 nucleotide, which occurs due to an extra unpaired base in the DNA sequence concerning sgRNA or an extra unpaired base in sgRNA for DNA sequence in the genome, respectively. From the treated and untreated stable cells, fibroblasts, and iPSC-RPE, gDNA was isolated and amplified using primers specific to off-target sites. All the primer sequences are listed in Supplemental Table 6. Deep-sequencing and data analysis were performed as described above.

### rhAmp off-target analysis in W53X mice.

As the gDNA yield from the mouse optic cup was too low to amplify all the off-target sites separately, we used a highly efficient RNase H2-dependent (rhAmp) PCR technique that can amplify different targets using a single PCR reaction. Amplification and sequencing were performed according to IDT rhAmp instructions. The rhAmpSeq CRISPR panel was designed using the IDT designing tool (https://www.idtdna.com/pages/tools/rhampseq-design-tool) for the potential off-targets of mW53X-sgRNA identified using Cas-OFFinder (Supplemental Table 7). The amplicon library was prepared using the rhAmpSeq CRISPR Library Kit (IDT, 10007317) and rhAmpSeq i5 and i7 index primers. The purified library was sequenced on the MiniSeq instrument from Illumina. Sequencing analysis was performed using the IDT rhAMP CRISPR analysis tool (https://www.idtdna.com/site/analysislab).

### Immunocytochemistry.

Kir7.1 protein expression was assessed in the pool of W53X-mutant, WT, and base-edited HEK293 stable cells by immunocytochemistry as described earlier (10). As the protein is GFP tagged, GFP mouse monoclonal primary antibody (Cell Signaling, 2955, 1:250) was used to enhance the Kir7.1 protein expression for its detection in the cells. Sodium potassium ATPase rabbit monoclonal primary antibody (Thermo Fisher, ST0533, 1:500) was used to label the cell membranes. Alexa Fluor 594–conjugated donkey anti-rabbit (Proteintech, SA00006.8, 1:500) and Alexa Fluor 488–conjugated donkey anti-mouse (Proteintech, SA00006.5, 1:500) secondary antibodies were used. DAPI was used as a nuclear counterstain. Immunostained cells were imaged on a confocal microscope (Nikon C2 Instruments).

### Electrophysiology assay.

A high-throughput automated patch clamp (Q Patch II, Sophion) was used to measure the whole-cell current from the HEK^WT^, HEK^W53X^, and base-edited HEK^W53X^ stable cells as described earlier (53). Briefly, the cells were grown in a T75 flask for 48 to 72 hours and then detached gently using Detachin. The cells were centrifuged at 90*g* for 1 minute and resuspended in serum-free medium containing 25 mM HEPES. The cells (3 M/mL) were kept on a shaker for 20 minutes before the experiment. Forty-eight cells were recorded in parallel on single-hole disposable Qplates with individual amplifiers. A pressure protocol was used to achieve cell positioning (–70 mbar), Giga seal (–75 mbar), and whole-cell configuration (5 pulses with –50 mbar increment between the pulses, first pulse of –250 mbar). The current was recorded in response to voltage-clamp steps from the holding potential (–10 mV) to voltages between –140 mV and +40 mV (Δ = 10 mV). More than 70% of the cells completed the experiment or run. The cells in which the stability was compromised during the experiment were judged by the leak current and excluded from the analysis. The extracellular solution contained the following: 135 mM NaCl, 5 mM KCl, 10 mM HEPES, 10 mM glucose, 1.8 mM CaCl_2_, and 1 mM MgCl_2_, pH adjusted to 7.4 and osmolarity 305 mOsm. The intracellular solution contained the following: 30 mM KCl, 83 mM K-gluconate, 10 mM HEPES, 5.5 mM EGTA, 0.5 mM CaCl_2_, 4 mM Mg-ATP, and 0.1 mM GTP, pH adjusted to 7.2 and osmolarity 280 mOsm. In an alternative external solution, NaCl was replaced with RbCl (140 mM) and used as an enhancer of Kir7.1 current. An extracellular solution with 20 mM Cs^+^ was used to block the Kir7.1 current. The data were analyzed using Sophion Analyzer, version 6.6.44. Whole-cell manual patch-clamp recording of base-edited hiPS-RPE cells was performed according to the standard protocol described elsewhere (7). There was no reporter or selection marker to aid in identifying the recipient or edited cells. Therefore, these cells were picked up randomly for the electrophysiology assay.

### Statistics.

The data analysis was done using Origin software (Origin 2020, OriginLab Corp.) and expressed as mean ± SEM. Two-tailed, unpaired Student’s *t* test was used to determine statistical differences. One-way ANOVA was used for multiple comparison, followed by Tukey’s honestly significant difference (HSD) method for adjustment of multiple comparisons. *P* < 0.05 was considered statistically significant.

### Study approval.

All work with LCA16 patient-derived cells (fibroblasts, iPSCs, and iPSC-RPE) was carried out following institutional, national, and international guidelines and approved by the University of Wisconsin–Madison’s Institutional Review Board and Stem Cell Research Oversight Committee. The animal protocols followed the ARVO Statement for use in ophthalmic and vision science research and were approved by the University of Wisconsin School of Medicine and Public Health Animal Care and Use Committee.

### Data availability.

Data are available in the paper’s supplemental material. Values associated with the main manuscript and supplemental material, including the values for all data points shown in graphs and values behind any reported means, are in the Supporting Data Values file. Additional data can be made available from the corresponding author upon request.

## Author contributions

MK and PKS drafted the manuscript. The shared co–first authorship is assigned based on the authors’ contributions to the study and drafting of the manuscript. MK, PKS, YW, DS, GAN, AAA, DMG, DRL, SG, KS, and BRP conceptualized the study. MK, PKS, YW, DS, GAN, AAA, DMG, DRL, SG, KS, and BRP devised the methodology. MK, PKS, YW, DS, AS, GAN, SS, YT, YC, and AAA carried out the investigation. MK, PKS, DMG, SG, KS, and BRP validated the study. MK, PKS, and YW carried out the formal data analysis. The data were visualized by MK, PKS, YW, DMG, SG, KS, and BRP. DS, GAN, YT, KLE, COT, and DRL provided the resources. MK, PKS, YW, GAN, DMG, DRL, SG, KS, and BRP curated the data. DMG, DRL, SG, KS, and BRP supervised the study. DMG, DRL, SG, KS, and BRP acquired funding.

## Supplementary Material

Supplemental data

Supporting data values

## Version 1 08/10/2023

In-Press Preview

## Version 2 10/02/2023

Electronic publication
