# Supplementary material for: Nonviral base editing of KCNJ13 mutation preserves vision in a model of inherited retinal channelopathy
Source: J Clin Invest. 2023 Oct 2;133(19):e171356. doi: 10.1172/JCI171356 (PMC10541187; doi:10.1172/JCI171356)
Supplement: Supplemental data [file jci-133-171356-s054.pdf]

Nonviral base editing of the retina *in vivo* preserves vision in a model of inherited channelopathy

Authors: Meha Kabra[1, 2, #], Pawan K. Shahi[1, 2, #], Yuyuan Wang[3, 4], Divya Sinha[2, 5], Allison Spillane[1], Gregory A. Newby[6, 7, 8], Shivani Saxena[2, 3, 4], Yao Tong[3, 4], Yu Chang[1], Amr A. Abdeen[2, 3, 4], Kimberly L. Edwards[2, 5], Cole O. Theisen[5], David R. Liu[6, 7,8], David M. Gamm[2,5, 9], Shaoqin Gong[2, 3, 4, 9, *], Krishanu Saha[1, 2, 3, 4, 10, *], Bikash R. Pattnaik[1,2,9, *]

Author affiliations: [1]Department of Pediatrics, University of Wisconsin-Madison, Madison, WI, United States. [2]McPherson Eye Research Institute, University of Wisconsin-Madison, Madison, WI, United States. [3]Department of Biomedical Engineering, University of Wisconsin-Madison, Madison, WI, United States. [4]Wisconsin Institute of Discovery, University of Wisconsin-Madison, Madison, WI, United States. [5]Waisman Center, University of Wisconsin-Madison, Madison, WI, United States. [6]Merkin Institute of Transformative Technologies in Healthcare, Broad Institute of Harvard and MIT, Cambridge, MA, United States. [7]Howard Hughes Medical Institute, Harvard University, Cambridge, MA, United States. [8]Department of Chemistry and Chemical Biology, Harvard University, Cambridge, MA, United States. [9]Department of Ophthalmology and Visual Sciences, University of Wisconsin-Madison, Madison, WI, United States. [10]Center for Human Genomics and Precision Medicine, University of Wisconsin-Madison, Madison, WI, United States.

# Equal contribution first authors
***Corresponding authors:** pattnaik@wisc.edu, ksaha@wisc.edu, shaoqingong@wisc.edu.
Bikash R. Pattnaik, PhD
University of Wisconsin-Madison
Office-SMI 112, Lab-SMI106
1300 University Avenue, Madison WI 53706
Phone: +1 6082659486 (Off), +16082620991 (Lab)
Email: pattnaik@wisc.edu

Supplementary Note

Inefficient repair of W53X in patient iPSC-RPE^{W53X} using CRISPR-Cas9 nuclease

We first assessed the correction of the homozygous *KCNJ13* W53X mutation in iPSC-derived RPE (iPSC-RPE$^{W53X/W53X}$) using Cas9 nuclease-mediated HDR. We delivered Cas9 nuclease and sgRNA via the lentiviral vector, LentiCRISPRv2-mCherry (Supplementary Figure 1), and the HDR donor template (ssODN-ATTO488) via SNC. The size and zeta-potential of donor templated-encapsulated SNC were summarized in Supplementary Table 1. We confirmed the delivery of both constructs using their cognate fluorescent reporters (Supplementary Figure 2A). Deep sequencing analysis of the treated samples indicated that most of the indels (5.05 ± 1.24%) were created downstream of the pathogenic mutation (TAG), resulting in no change to the reading frame (Supplementary Figure 2, B and C). Only a small fraction of reads showed in-frame indel formation (1.03 ± 0.52%) and a corrected wildtype (WT) genotype (0.34%) (Supplementary Figure 2D), both of which are predicted to remove (i.e., repair) the W53X stop codon during translation of the edited *KCNJ13* mutant allele. Although the green fluorescence in the treated cells showed a successful delivery of ssODN, we did not observe the inclusion of any silent nucleotide bases from the ssODN (Supplementary Figure 2B). Most reads (94.50 ± 1.25%) were unedited in the treated cells.

Next, we measured the Kir7.1 channel function in gene-edited cells expressing the red (mCherry) and green (ATTO488) fluorescence, indicating that they had received both Cas9-sgRNA and ssODN. Single-cell patch-clamp recordings revealed a normal Kir7.1 current with an inward current of -101.1 ± 35.54 pA at -150 mV in treated cells. Substitution of extracellular Na^+ with rubidium (Rb^+), a known activator of Kir7.1, increased the Kir7.1 inward current by 7-fold to -713.5 ± 92.97 pA (Supplementary Figure 2, E-G). These results indicate that delivery of both Cas9-sgRNA and ssODN can sometimes generate function-restoring gene edits. However, the exact nature and frequency of editing outcomes in the single cells we recorded by patch clamp could not be assayed due to the technical challenges of amplifying genomic DNA from a single cell.

Altogether, this nuclease-mediated HDR approach did result in a very small frequency of functional RPE cells– indicating that gene correction is a viable strategy to rescue Kir7.1 function in RPE cells. However, the low efficiency and high heterogeneity of the edits diminished the translational potential of this strategy.

Supplementary tables

Supplementary Table 1: Size and zeta-potential of ATRA-modified SNCs with different payloads

Payload	Size (D_h, nm)	Zeta-potential (mV)
ssODN donor template	46±4	3.6±1.6
ABE mRNA+sgRNA	42±4	4.9±1.8

Supplementary Table 2: Primers for in-fusion cloning of *KCNJ13* in FLP-In™ expression vector

**Primer name**	**Sequence (5'-3')**	**GC %**
In-fusion FP	TCACTATAGGGAGACCCAAGCTGGCTAGCGTTTAAACTTAatggtgagcaagggcgagga	50.0
In-fusion RP	AGTCGAGGCTGATCAGCGGGTTTAAACGGGCCCTCTAGACttattctgtcagtcctgttt	50.0
GFP FP	CAAGTCCGGACTCAGATCTCGAGCTC	57.1
Kir7.1 RP	TTATTCTGTCAGTCCTGTTT	72.7

FP: Forward primer, RP: Reverse primer. Primers for in-fusion cloning were designed using the Gibson assembly primer design tool available at https://tools.sgidna.com/gibson-assembly-primers.html and ordered from IDT (https://www.idtdna.com). The homology sequence is in uppercase, and the annealing sequence is in lowercase. The primers for Sanger sequencing (GFP FP and Kir7.1 RP) were designed using the NCBI Primer-BLAST tool (https://www.ncbi.nlm.nih.gov/tools/primer-blast/).

Supplementary Table 3: Primers to genotype mice

Primer name	Sequence (5'-3')	GC %
mKcnj13-FP	TAAATCAGCTACGGGCTAACA	42.86
mKcnj13-RP	CTGTGATAAAAGCCTCTAGCA	42.86

The primers were designed using the NCBI Primer-BLAST tool (https://www.ncbi.nlm.nih.gov/tools/primer-blast/). Annealing temperature 55 °C.

Supplementary Table 4: Primers to amplify the *hKCNJ13* on-target sites

**Primer name**	**Sequence (5'-3')**	**GC %**
NGS-hKCNJ13-FP	TCAAATGGATGGCGCTCAAAGA	45.0
NGS-hKCNJ13-RP	ATACCAGAGCACTGCAAAGACAA	43.0

The primers were designed using the NCBI Primer-BLAST tool and ordered with an adaptor sequence for the Illumina NGS platform from IDT. Adaptor sequence for FP: 5'-ACACTCTTTCCCTACACGACGCTCTTCCGATCT-3'Adaptor sequence for RP: 5'-GTGACTGGAGTTCAGACGTGTGCTCTTCCGATCT-3'.

Supplementary Table 5: Putative off-targets sites of human-sgRNA identified using Cas-OFFinder

S. No.	Location	Chr	strand	mismatches	G	C	G	C	T	A	G	C	G	T	T	G	G	A	T	G	A	T	G	T	T	G	G
1	STK24	13	-	3	·	T	·	·	·	·	·	·	C	·	·	·	·	·	·	·	T	·	·	·	G	G	G
2	GRCh38:133058732 Intergenic	10	-	3	·	·	A	·	G	·	·	G	·	·	·	·	·	·	·	·	·	·	·	·	T	A	G
3	GRCh38:50530925 Intergenic	18	-	2	·	G	·	·	·	·	C	·	·	·	·	·	·	·	·	·	·	·	·	·	C	A	G
4	PPM1K Intronic	4	+	3	·	·	·	A	G	·	·	·	T	·	·	·	·	·	·	·	·	·	·	·	T	G	G
5	AP2B1 Intronic	17	-	4	·	·	·	·	·	T	·	·	T	·	·	·	T	·	·	·	·	·	·	·	G	G	G
6	TMEM 117 Intronic	12	+	4	·	·	.	.	·	T	T	·	T	·	·	T	·	·	·	·	·	·	·	·	T	G	G
7	GRCh38:113728054 Intronic	4	+	4	·	·	·	G	·	·	·	A	·	·	G	·	·	·	G	·	·	·	·	·	G	G	G
8	HRH1 Intronic	3	-	4	·	·	·	·	·	G	·	A	·	·	·	·	T	·	·	·	C	·	·	·	G	G	G
9	GRCh38:55352815 Intergenic	12	-	3	·	·	·	·	·	·	·	·	T	A	·	·	·	T	·	·	·	·	·	·	T	G	G

Supplementary Table 6: Primers to amplify the off-target sites of human-sgRNA

S. No.	Primer name	Sequence (5'-3')	GC %
1	STK24_NGS_F	GGGATGCCACTTGGAGAACT	55.0
	STK24_NGS_R	ATTCTGGGTACACACTCCCA	50.0
2	ING_CHR10_NGS_F	CAGAGAGCTCCTTCTTTCTCTGA	47.83
	ING_CHR10_NGS_R	AAGCTCCTTCCCCAAGCAAA	50.0
3	ING_CHR18_NGS_F	TGTAATGGTGATCTAGTCACAGAG	41.67
	ING_CHR18_NGS_R	GCCTCATTCTGAAAGGGTCC	55.0
4	PPM1K_NGS_F	CCACTGCAGGTAGAGCTGTT	55.0
	PPM1K_NGS_R	CTGCACTCAAGCTGGGTTTC	55.0
5	AP2B1_NGS_F	TGAGCTCTTCCTGTAAGTGACC	50.0
	AP2B1_NGS_R	TGCATACCTTTGATGGCCTG	50.0
6	TMEM117_NGS_F	GTAGGTTCAATTTCTAACCCTTGC	55.0
	TMEM117_NGS_R	AGAGGAGAAATAGGAAGCAAAGT	55.0
7	INTRONIC_CHR4_NGS_F	TGAAGTCCAAGAAAAGGCAAA	38.0
	INTRONIC_CHR4_NGS_R	CCTCCCCAAACTGAATACAAAA	41.0
8	HRH1_NGS_F	GGGTACATGGCTATTGAGTAGG	50.0
	HRH1_NGS_R	GCCACCAGTTATGGCTCACT	55.0
9	ING_CHR12_NGS_F	CATGATAACTGTGGTGCGCT	50.0
	ING_CHR12_NGS_R	GTGACCTAAATCAGTTGGATGGAG	45.83

The primers were designed using the NCBI Primer-BLAST tool and ordered with an adaptor sequence for the Illumina NGS platform from IDT. Adaptor sequence for FP: 5'-ACACTCTTTCCCTACACGACGCTCTTCCGATCT-3'Adaptor sequence for RP: 5’-GTGACTGGAGTTCAGACGTGTGCTCTTCCGATCT-3'.

Supplementary Table 7: Potential off-target sites of mouse W53X-sgRNA identified using Cas-OFFinder

S.No	#Bulge type	crRNA	DNA	Chr	Position	Strand	Mismatches	Bulge Size
1	DNA	GCGCTAGCGCTGGAT-GATGCNRG	GCaCTAGgGCTGGATGGATGCAAG	chr5	27794505	-	2	1
2	X	GCGCTAGCGCTGGATGATGCNRG	GgtCcAGCGCaGGATGATGCTGG	chr2	30011064	+	4	0
3	RNA	GCGCTAGCGCTGGATGATGCNRG	GgGCgAGC-CTGGATGATGCTGG	chr2	74762664	-	2	1
4	RNA	GCGCTAGCGCTGGATGATGCNRG	GttCTAGC-CTGGATGATGCAAG	chr2	116880781	-	2	1
5	X	GCGCTAGCGCTGGATGATGCNRG	GCtCaAGCtCTGGtTGATGCCAG	chr17	47011867	+	4	0
6	X	GCGCTAGCGCTGGATGATGCNRG	GgGCTAGCGCTGGATGcTGCTGG	chr16	38496875	-	2	0
7	X	GCGCTAGCGCTGGATGATGCNRG	GCcCTcGgGaTGGATGATGCAAG	chr9	103261947	+	4	0
8	X	GCGCTAGCGCTGGATGATGCNRG	GtGCTAtCtCTaGATGATGCCAG	chr6	134150494	+	4	0
9	X	GCGCTAGCGCTGGATGATGCNRG	GCGCTAGCcCTGGATGgTGgTGG	chr11	97642214	-	3	0
10	X	GCGCTAGCGCTGGATGATGCNRG	GCtaTgGtGCTGGATGATGCTGG	chr3	28240364	+	4	0
11	DNA	GCGCTAGCGCT-GGATGATGCNRG	GCGCTAGCGCTGGGAgGgTGCTGG	chr7	93020838	-	2	1

Supplementary Table 8: sgRNA design using CRISPR-RGEN and PnB Designer

CRISPR Target (5'-3')	PAM	AA sequence	Direction	GC contents (% w/o PAM)
AACGCTAGCGCATGTCCATT	AGG	CAG<br>Q	-	50.0
TAATGGACATGCGCTAGCGT	TGG	ATGGGC<br>M G	+	50.0
GCGCTAGCGTTGGATGATGT	TGG	TGG<br>W	+	50.0

The sgRNAs were designed using CRISPR-RGEN tool available at http://www.rgenome.net. The letters highlighted in red are the targeted nucleotide base using the protospacer. Amino acids listed in blue would create a missense mutation, while those in green would produce the desired amino acid change to make a WT Kir7.1 protein.

Protospacer	PAM	EditPos.	Base.Editor
GCGCTAGCGTTGGATGATGT	TGG	6	ABEmax/ABE8e
ATGCGCTAGCGTTGGATGAT	GTTGGT	8	SaKKH-ABEmax/ABE8e

The sgRNAs were designed using PnB Designer available at https://fgcz-shiny.uzh.ch/PnBDesigner/. Letters highlighted in red are the targeted nucleotide base using the protospacer and BE.

Supplementary Table 9: sgRNA design for mouse *Kcnj13* using Benchling.

Strand	sgRNA	PAM	Purpose	On-target score	Off-Target score
+	GAATCCTAATGGACATGCGC	TGG	WT disruption	56.6	48.4
+	GCGCTAGCGCTGGATGATGC	TGG	W53X editing	57.1	82.9

Supplementary Figures

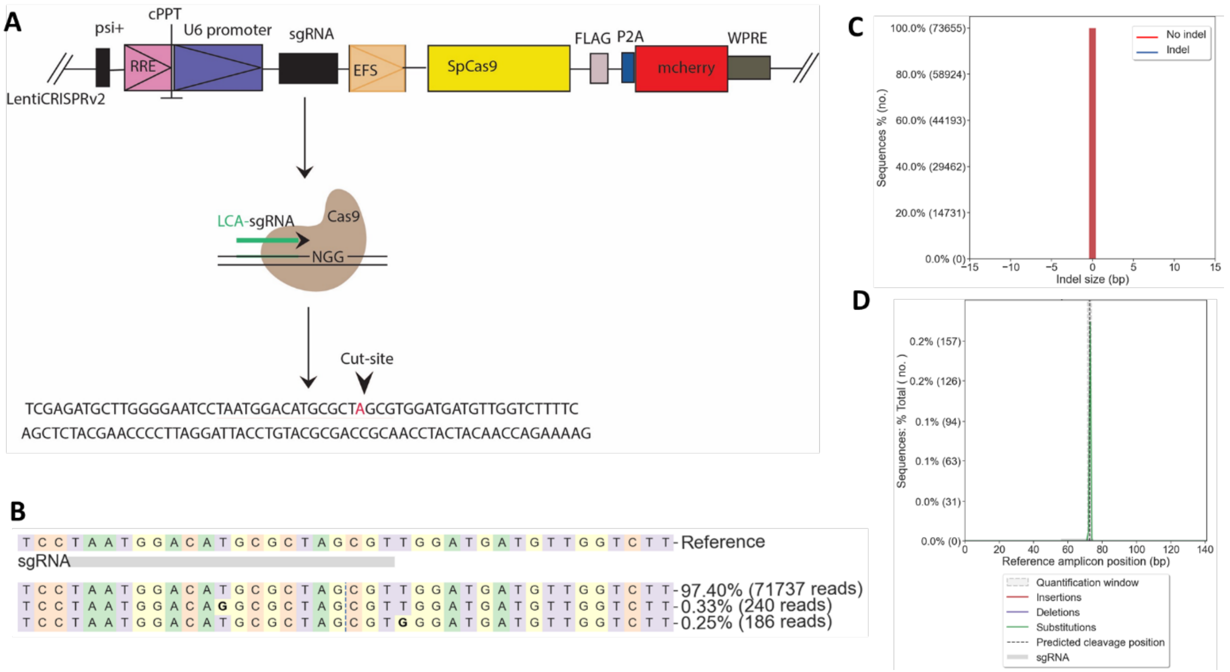

Supplementary Figure 1: Gene editing strategy and sequencing readouts from untreated iPSC RPE^{W53X}. *A] Construct design of lentiviral CRISPRv2 vector demonstrating the sgRNA and spCas9 location. B] Nucleotide distribution around the cut site (vertical dashed line) of sgRNA. C] Indel size distribution in untreated samples. D] Mutation position distribution showing the editing profile in untreated samples.*

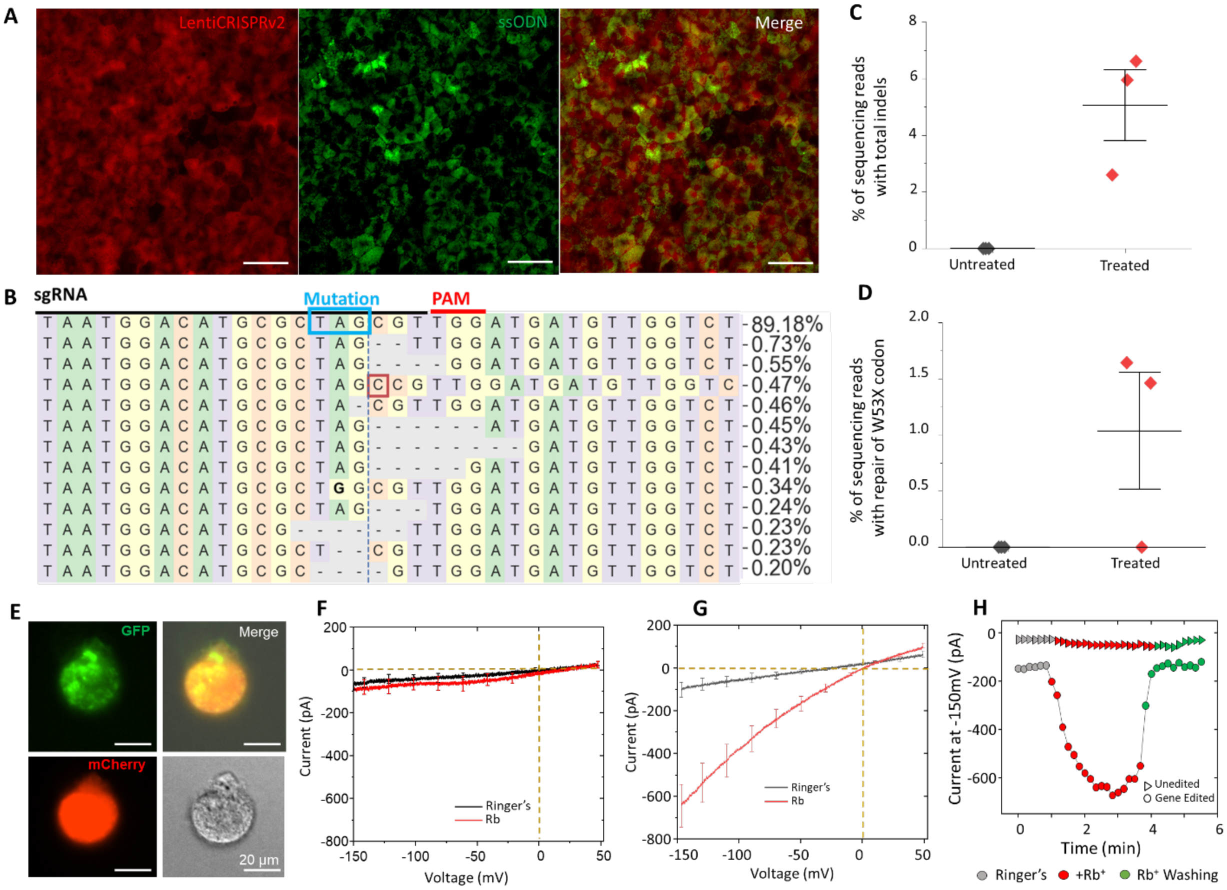

Supplementary Figure 2: CRISPR-Cas9 nuclease editing outcomes in iPSC-RPE$^{W53X/W53X}$. (A) *Transduction with LentiCRISPRv2 –mCherry carrying Cas9 and sgRNA (red panel) and delivery of ssODN-ATTO488 via SNCs (green panel) in iPSC RPE$^{W53X/W53X}$ showing the dual fluorescence (merged panel). Scale 50 µm.* ***(B)*** *Deep sequencing reads from cells that received both LentiCRISPRv2 and ssODN treatment show the nucleotide distribution around the DSB cleavage site (vertical dashed line). The sgRNA protospacer location is highlighted by a black line, the protospacer adjacent motif (PAM) by a red line, and the pathogenic early stop codon (TAG) mutation in the blue box. Substitutions are highlighted in bold, insertions are shown in red boxes, and a dash shows deletions.* ***(C)*** *Total percentage of sequencing reads containing indels at the DSB site across the treated samples (n=3, biological replicates).* ***(D)*** *Percentage of reads comprised of WT reads and in-frame indels observed in the treated samples (n=3, biological replicates).* ***(E)*** *Fluorescence microscopy image of a single dissociated iPSC-RPE cell expressing both reporters, chosen for manual patch-clamping. Scale 20 µm.* ***(F)*** *Average current-voltage plot from untreated cells used as a reference. The black line indicates the current recorded in HEPES 'Ringer's (HR) solution and red line indicates the current recorded in the presence of extracellular Rb^{+} ion. (n=3).* ***(G)*** *Average current-voltage plot demonstrating K^{+} current following gene editing. The grey line indicates the current recorded in the HR solution, while the red line indicates the current measurement in the presence of the Kir7.1 channel current enhancer, Rb^{+} ion (n=4).* ***(H)*** *A representative time course illustrating the reversible increase in inward current by extracellular (HR, grey), Rb^{+}(red), and wash (green).*

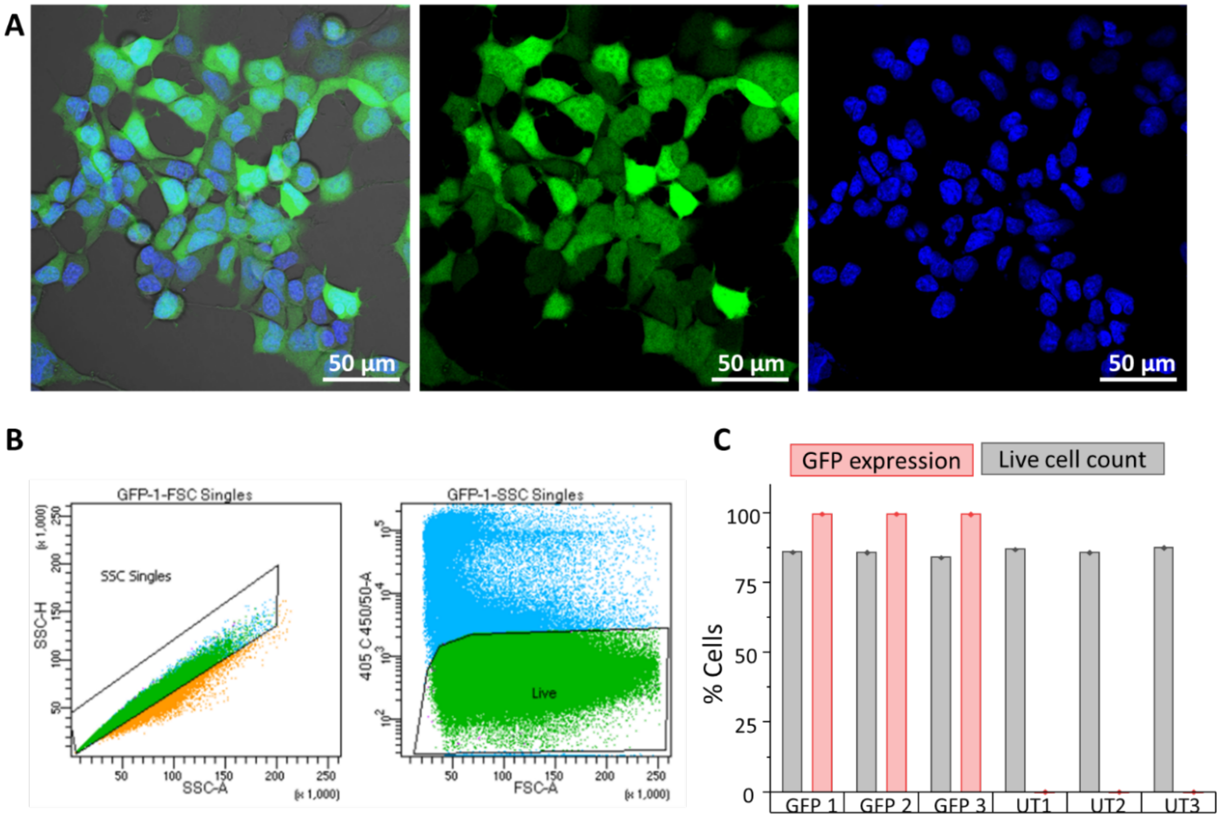

Supplementary Figure 3: Electroporation efficiency in HEK293 cells. *A] Confocal imaging of HEK293 cells electroporated with GFP mRNA showing the efficient delivery of GFP (green). Hoechst stain (blue) was used to label nuclei. B] Fluorescence-activated cell sorting (FACS) of GFP-electroporated cell population. Single cell population by forward scatter (FSC) and side scatter (SSC). C] % of live and GFP positive cells from a cell population either electroporated with GFP (n=3) or placebo treated (n=3).*

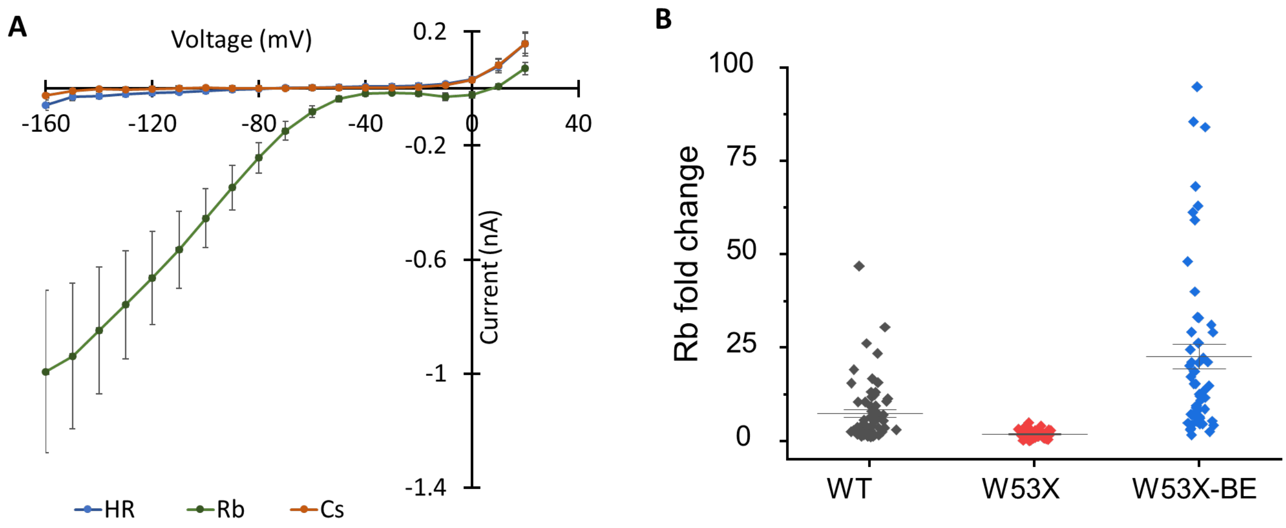

Supplementary Figure 4: Automated patch-clamp recordings from HEK$^{W53X/W53X}$, HEKT$^{WT/WT,}$ and base-edited HEK^{W53X} cells. *A] I-V curve for HEK$^{WT/WT}$ cells (n=3) showing a large negative membrane potential. B] The fold changes for Rb^{+} in HEK$^{WT/WT}$, HEK$^{W53X/W53X}$, and base-edited HEK^{W53X} cells.*

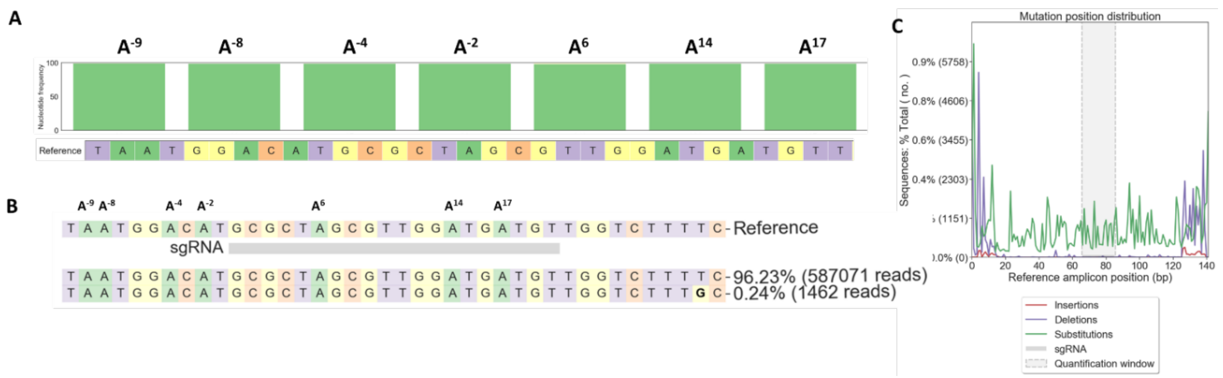

Supplementary Figure 5: Sequencing readouts from untreated LCA16-Fibroblasts^{W53X} used as reference. *A] Nucleotide distribution around sgRNA location as observed in sequencing reads. B] Percentage of sequencing reads observed in the untreated sample. C] Percentage distribution of substitution and deletion at sgRNA location.*

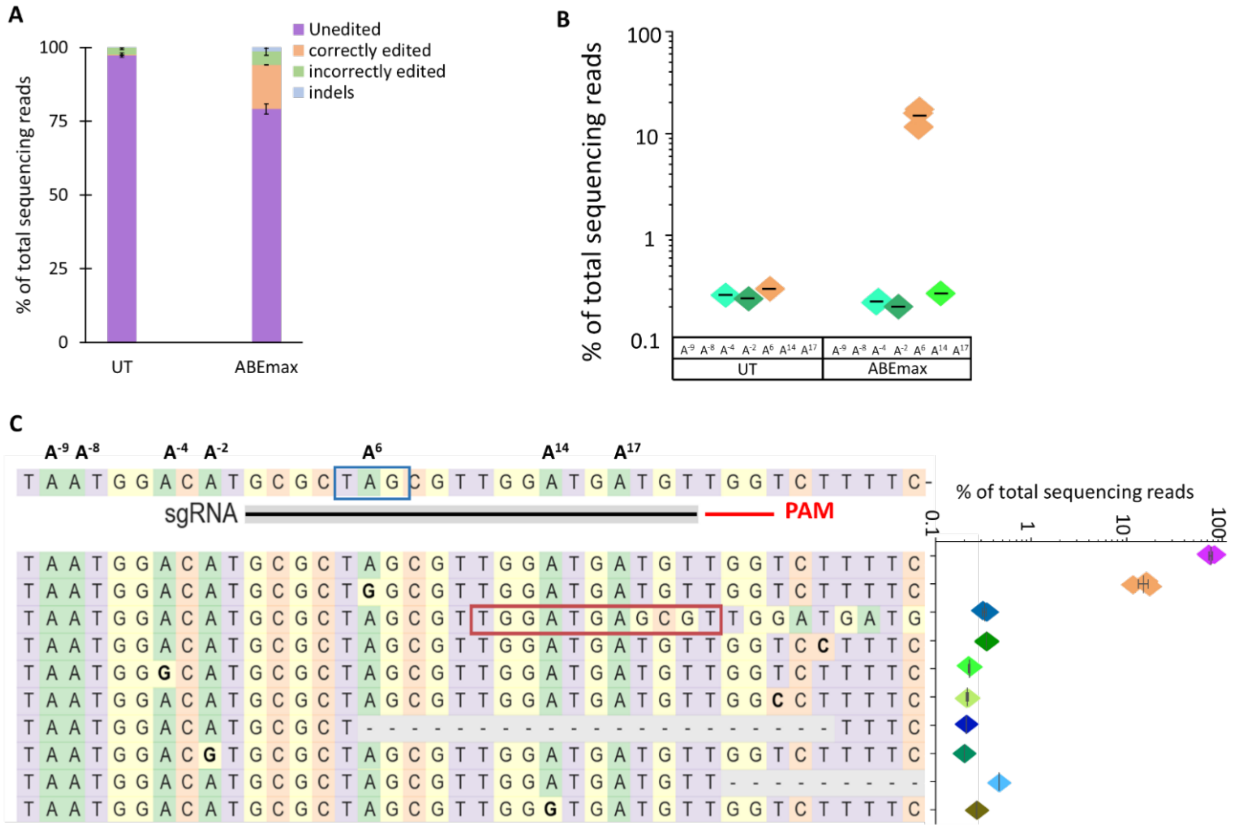

Supplementary Figure 6: Evaluation of ABE7.10max mRNA + sgRNA combinations to correct the W53X allele in Fibro^{W53X}. *A] Base editing efficiencies shown as the % of total DNA sequencing reads, classified as unedited, correctly edited, incorrectly edited due to bystander 'A' edits, and with indels in treated and untreated (UT) cells. B] % Editing of the target (A^{6}) and bystander (A^{-9}, - A^{-8}, A^{-4}, A^{-2}, A^{14}, A^{17}) 'A' to 'G' as observed in three independent experiments. C] The sgRNA location is marked by black line, PAM by red line, and mutation in the blue box. All the 'A' bases within the protospacer are numbered from 1-20 based on their location. The 'A' bases downstream of the protospacer are numbered from -1 to -9, considering +1 as the first base of the protospacer. The top 10 most frequent alleles generated by ABE7.10max mRNA treatment show the nucleotide distribution around the cleavage site for sgRNA. Substitutions are highlighted in bold, insertions are shown in the red box, and deletions are shown by dashes. The scatter plot shows the frequency of reads observed in treated cells (n=3 biological replicates). Figures presenting data from replicates are shown as mean ± SEM.*

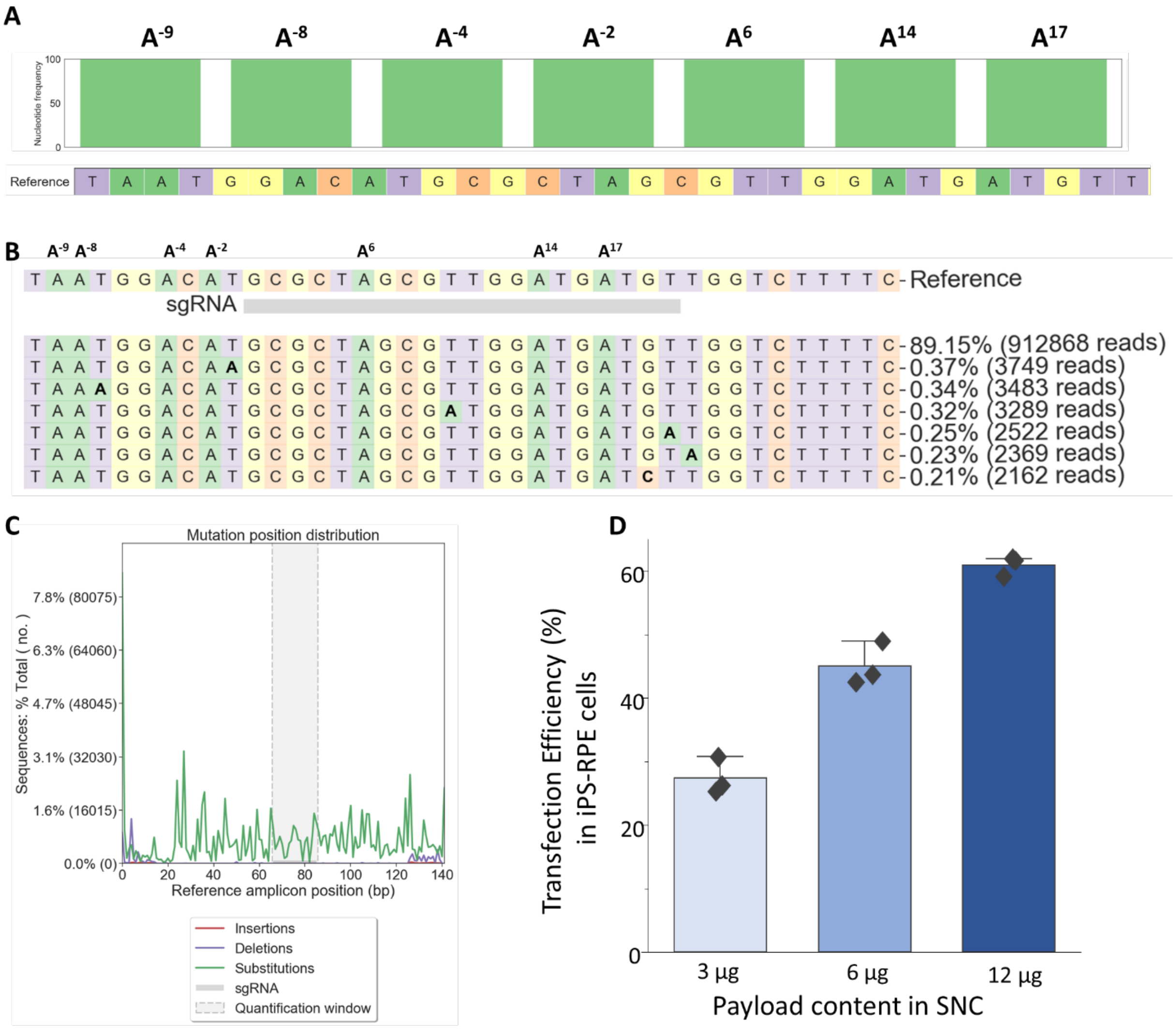

Supplementary Figure 7: Sequencing readouts from untreated iPSC RPE^{W53X} cells used as reference. *A] Nucleotide distribution around sgRNA location as observed in sequencing reads. B] Percentage of sequencing reads observed in the untreated sample. C] Percentage distribution of substitution and deletion at sgRNA location. D] Transfection efficiency in iPSC-RPE cells using SNCs.*

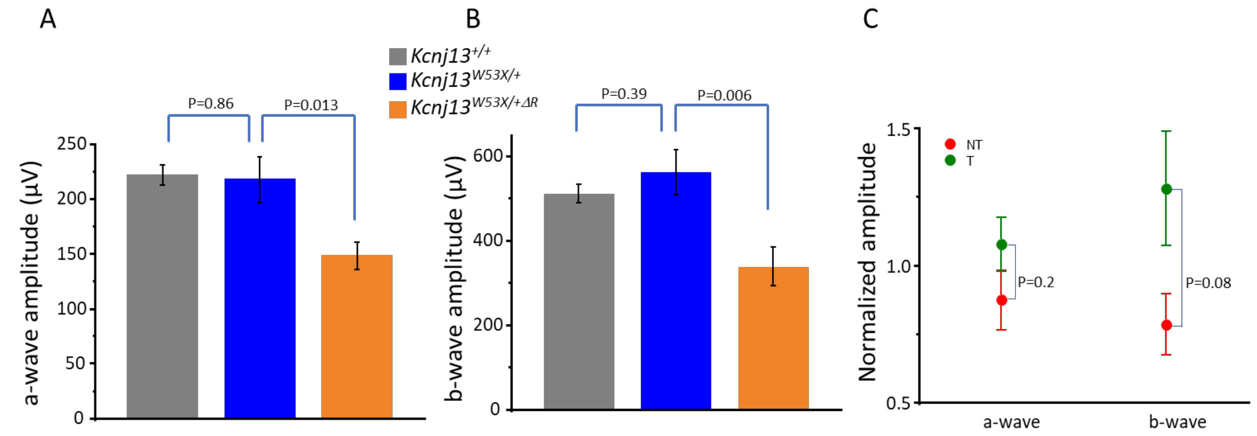

Supplementary Figure 8: Comparison of a-wave and b-wave amplitude*: A] a-wave B] b-wave amplitude comparison after scotopic ERG on Kcnj13$^{+/+}$, Kcnj13$^{W53X/+}$ and Kcnj13$^{W53X/+\Delta R}$ mice. C] comparison of a-wave and b-wave amplitudes in Kcnj13$^{W53X/+\Delta R}$ following the injection of base editor.*

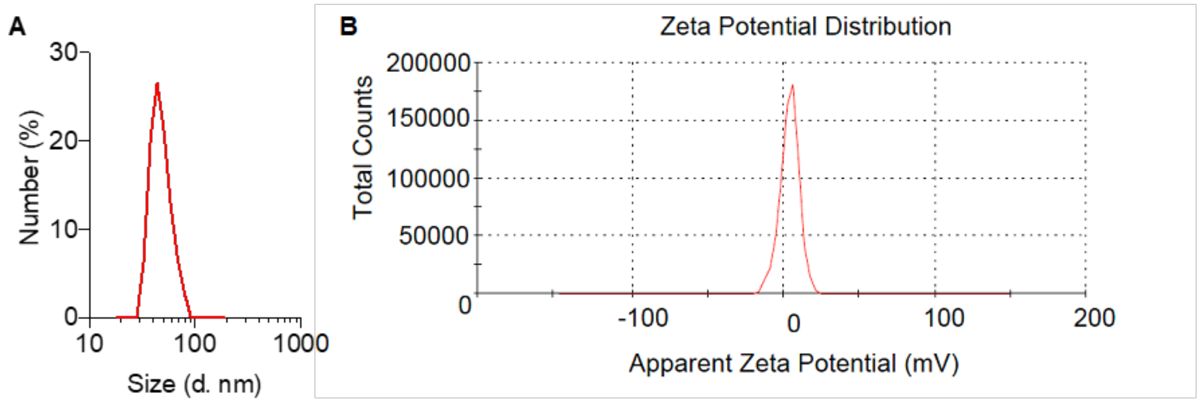

Supplementary Figure 9: A] *Size distribution and* ***B]*** *Zeta-potential of ABE mRNA+sgRNA-encapsulated SNC with ATRA modification.*

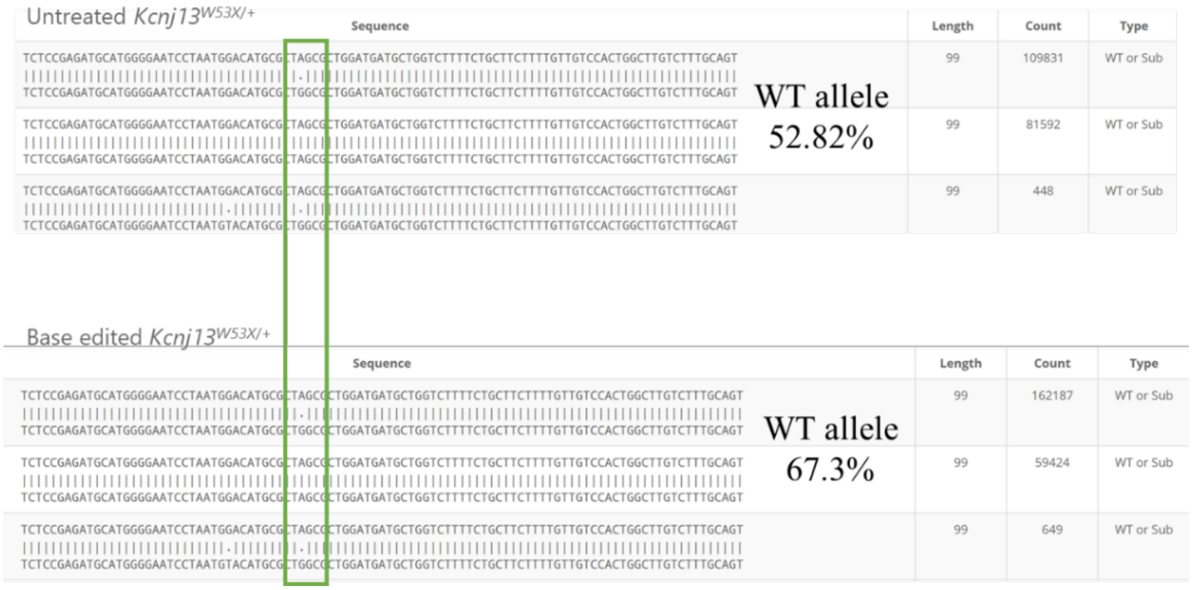

Supplementary Figure 10: Deep sequencing reads from the edited fibroblasts isolated from Kcnj13$^{W53X/-}$ mice. *The sequencing reads were generated by editing mouse W53X alleles within fibroblasts using ABE8e and W53X-sgRNA, delivered via nucleofection.*

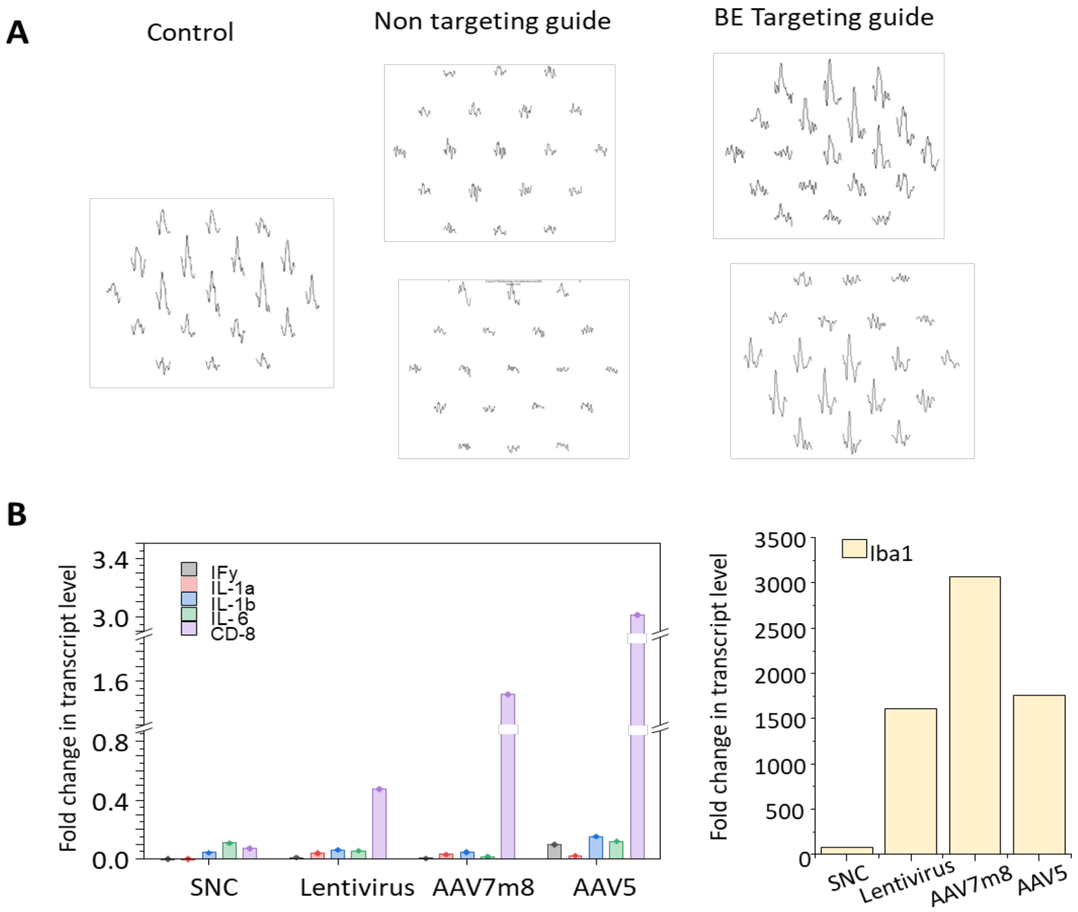

Supplementary Figure 11: *A] mfERG measurements from the control mice (left panel), after wildtype allele disruption (middle panel) and after injection of base editor to Kcnj13$^{W53X/+\Delta R}$ mice (right panel)***.** *B] Level of inflammatory cytokine transcripts triggered by SNC, Lentivirus, AAV7m8, and AAV5.*

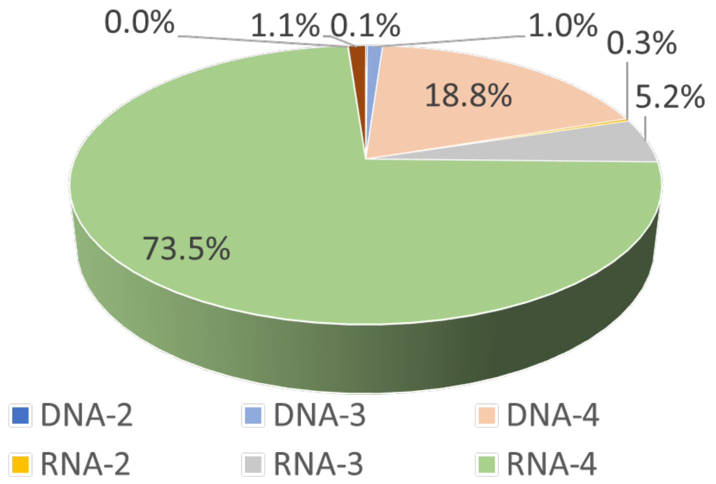

Supplementary Figure 12: Genomic off-target sites for human W53X sgRNA*. The sites were identified using standard criteria, up to 1-4 mismatches and DNA/ RNA bulge (size=1 nucleotide).*

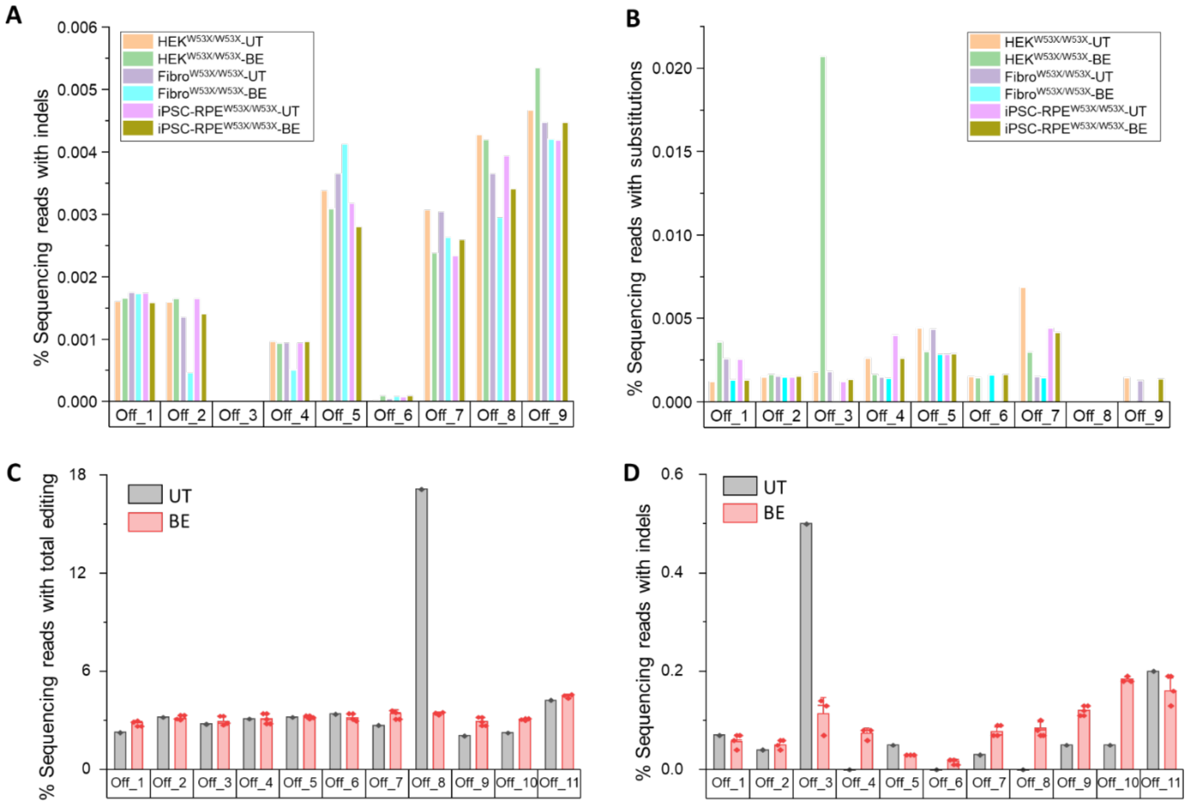

Supplementary Figure 13: Off-target editing analysis of the base editor delivered by SNC in the human in vitro LCA16 model and mouse in vivo LCA16 model. (A) *Percentage of sequencing reads with indels as observed by deep sequencing analysis in Base editor treated (BE) HEK^{W53X}, Fibro^{W53X,} and iPSC-RPE$^{W53X/W53X}$ and their respective untreated (UT) cells (H stands for HEK293 stable cells, L stands for LCA16 fibroblasts and R stands for iPSC-RPE).* ***(B)*** *Percentage of sequencing reads with substitutions as observed by deep sequencing analysis in BE-treated HEK^{W53X}, Fibro^{W53X}, iPSC-RPE$^{W53X/W53X,}$ and their respective untreated cells.* ***(C)*** *Percentage of sequencing reads with total editing including substitutions and NHEJ as observed by deep sequencing analysis of optic cup gDNA from ABE8e treated Kcnj13$^{W53X/+}$ mice (n=3 eyes) compared to a negative control Kcnj13$^{W53X/+}$mouse treated only with PBS (n=1 eye).* ***(D)*** *Percentage of sequencing reads with only indels as observed by deep sequencing analysis in ABE8e treated Kcnj13$^{W53X/+}$ (n=3 eyes) compared to a negative control Kcnj13$^{W53X/+}$mouse treated only with PBS.*

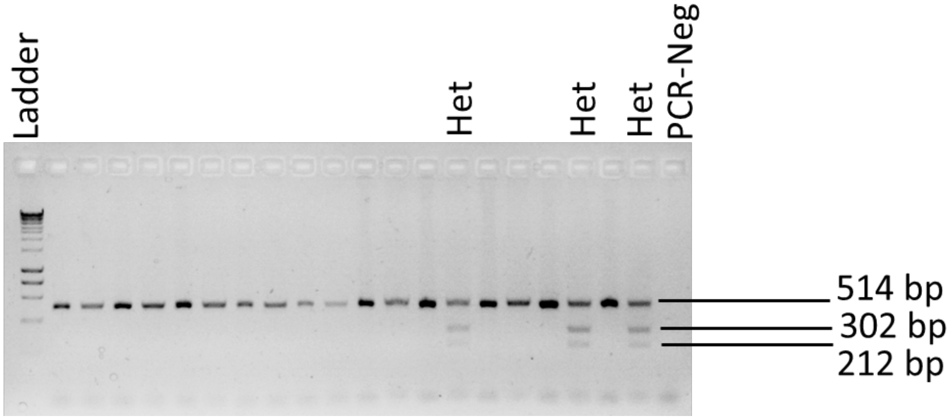

Supplementary Figure 14: Agarose gel electrophoresis showing the differences in W53X heterozygous and WT mice. *W53X mutation creates a restriction site for NheI; therefore, the W53X allele resulted in two (212 bp and 302 bp) fragments while the WT allele only one (514 bp) fragment.*
